# Design of optimal nonlinear network controllers for Alzheimer's disease

**DOI:** 10.1371/journal.pcbi.1006136

**Published:** 2018-05-24

**Authors:** Lazaro M. Sanchez-Rodriguez, Yasser Iturria-Medina, Erica A. Baines, Sabela C. Mallo, Mehdy Dousty, Roberto C. Sotero, on behalf of The Alzheimer’s Disease Neuroimaging Initiative

**Affiliations:** 1 Biomedical Engineering Graduate Program, University of Calgary, Calgary, Alberta, Canada; 2 Department of Radiology and Hotchkiss Brain Institute, University of Calgary, Calgary, Alberta, Canada; 3 Department of Neurology & Neurosurgery, McConnell Brain Imaging Centre, Montreal Neurological Institute, Montreal, Quebec, Canada; 4 Ludmer Centre for NeuroInformatics and Mental Health, Montreal, Quebec, Canada; 5 Faculty of Medicine and Dentistry, University of Alberta, Edmonton, Alberta, Canada; 6 Departament of Developmental Psychology, University of Santiago de Compostela, Santiago de Compostela, Spain; Oxford University, UNITED KINGDOM

## Abstract

Brain stimulation can modulate the activity of neural circuits impaired by Alzheimer’s disease (AD), having promising clinical benefit. However, all individuals with the same condition currently receive identical brain stimulation, with limited theoretical basis for this generic approach. In this study, we introduce a control theory framework for obtaining exogenous signals that revert pathological electroencephalographic activity in AD at a minimal energetic cost, while reflecting patients’ biological variability. We used anatomical networks obtained from diffusion magnetic resonance images acquired by the Alzheimer’s Disease Neuroimaging Initiative (ADNI) as mediators for the interaction between Duffing oscillators. The nonlinear nature of the brain dynamics is preserved, given that we extend the so-called state-dependent Riccati equation control to reflect the stimulation objective in the high-dimensional neural system. By considering nonlinearities in our model, we identified regions for which control inputs fail to correct abnormal activity. There are changes to the way stimulated regions are ranked in terms of the energetic cost of controlling the entire network, from a linear to a nonlinear approach. We also found that limbic system and basal ganglia structures constitute the top target locations for stimulation in AD. Patients with highly integrated anatomical networks–namely, networks having low average shortest path length, high global efficiency–are the most suitable candidates for the propagation of stimuli and consequent success on the control task. Other diseases associated with alterations in brain dynamics and the self-control mechanisms of the brain can be addressed through our framework.

## Author summary

This work aims to close the knowledge gap between theory and experiment in brain stimulation. Previous modeling approaches for stimulation have overlooked the nonlinear dynamical nature of the brain and failed to shed light on efficient mechanisms for the exogenous control of the brain. Amid the current efforts for developing personalized medicine, we introduce a framework for producing tailored stimulation signals, based on individual neuroimaging data and innovative modeling. This is the first time, to our knowledge, that brain stimulation for the most common cause of dementia, Alzheimer’s disease, is theoretically addressed. Our approach leads to the identification of potential target regions and subjects to successfully respond to brain stimulation therapies and yields various disease-reverting signals. Although focused on Alzheimer’s in this study, our methodology could be applied to other clinical conditions characterized by abnormalities in brain dynamics, like epilepsy and Parkinson’s, the treatment of which can benefit from the use of optimal control strategies.

## Introduction

Alzheimer’s disease (AD) is the most common cause of dementia, with classic biomarkers including vascular and glucose metabolism dysregulation, amyloid-β and tau deposition, white matter degeneration, functional impairment, and grey matter atrophy [1]. Because of the complex mechanisms and non-physiological factors that interact in an intricate manner [2], our understanding of the disease and ability to produce efficient therapeutic interventions has been limited.

One area of therapeutic interventions currently being investigated is brain stimulation, which aims to correct (control) pathological activity by steering it towards a trajectory (pattern of brain activity) we consider healthy. Several attempts have been made to assess the potential brain stimulation has for treating AD. For example, cognitive improvement was found immediately after applying repetitive transcranial magnetic stimulation (rTMS) and transcranial direct current stimulation (tDCS) [3]. An ongoing clinical trial has reported reversion of impaired glucose metabolism in the temporal and parietal association cortices along with slowing of cognitive indicators for the progression of AD by applying deep brain stimulation (DBS) in the fornix [4,5]. However, unlike the case of Parkinson’s disease where a decrease in tremor constitutes a short-term measure for the success of the therapy, these AD studies lacked such a biomarker. Consequently, they were unable to guarantee that their stimulation parameters were the optimal for their purposes. By optimal, we understand inputs that make the pathological state disappear while the energy used by the external controlling agent (cost) is minimal. Beyond the use of DBS for AD, most brain stimulation protocols are likely suboptimal in their selection of the signal shape, amplitude, and stimulation sites, since they are set by trial-and-error. Additionally, stimulation treatments are currently identical for all individuals with the same clinical condition, disregarding biological variability [4–8], and highlighting the need for an increased understanding on how to optimize stimulation protocols for individual patients. To address some of these issues, computational modeling techniques have been previously used [9–12].

However, modeling brain stimulation requires the consideration of some well-known facts, such as that the evolution of brain activity is intrinsically related to the subjacent anatomical network and the interplay of various neuronal populations [13]. As in any other network, it is reasonable to assume some elements (or nodes) have an architectural leading role in the self-regulation of that neural system [14,15]. An input feeding into one of these elements has the potential to propagate through the network, influencing the system towards the state desired by the controller. The existence and characteristics of such input signals are then given by the dynamical structure of the system and the way its elements are coupled to the inputs. Systems in which those signals that drive the activity to a desired configuration exist are known as controllable (as opposed to uncontrollable), relating to the property ‘controllability’ [16].

Some studies have focused on identifying the most suitable sites for network controllability from a structural viewpoint only [17] while simplifying the dynamical interactions occurring on top of the connectivity scaffold. Other studies [9–12] used linear dynamics to model neural processes, which are known to be intrinsically nonlinear [13,18–20]. Hence, their predictions on neural network control should be taken with caution. Neglecting the nonlinear nature of the brain for the sake of mathematical simplicity might bias or corrupt the results therein obtained. Taylor et al., in their seminal paper [10], simulated seizure abatement in a nonlinear model by means of the so-called pseudospectral method. Their approach assumed that the entire cortex was stimulated, thus resulting in a readily controllable system. However, a global control-strategy might be not achievable in practice, and may relate to high energy deposition over the brain tissue. Additionally, only stimuli that were independent of the state variables (open-loop) were considered, whereas recent evidence supports the idea of enhanced benefits associated with closed-loop brain stimulation [21–24].

Finally, the identification of optimal (electromagnetic) signals among the universe of those that can be created [6–8] has a paramount importance in terms of patient’s welfare and technological improvement. As such, modeling approaches should be able to predict the brain structures that better respond to targeted stimulation for achieving a control objective over the network. For instance, the surgical implantation of devices (for DBS) could be avoided if theoretical calculations envisage that stimulation of cortical neuronal conglomerates, with, e.g., tDCS, produces comparable results to what is achieved by means of DBS. In the same way, pinpointing optimal control signals likely translates to less exposure for the patient and to a reduction of procedure-related costs in terms of number of sessions required, the shape and amplitude of the signals that are used, etc.

In this work, we attempt to reconcile the theory of neural network control and the true nonlinear nature of the brain and shed light on the development of efficient stimulation therapies for AD. One framework that deals with nonlinearities while optimizing input signals for controlling dynamical systems is the state-dependent Riccati equation control (SDRE) [25,26]. SDRE has several applications in mechanical problems and aerospace engineering [26] though few in the fields of biological and high-dimensional systems, where the above-mentioned simplistic linear approaches have been preferred. We use SDRE to obtain the optimal signals to steer AD systems towards healthy states and to predict the best candidate brain regions and subjects to undergo a stimulation therapy.

We compute anatomical connection density matrices for the interaction between neuronal populations in cortical and subcortical brain regions from diffusion weighted magnetic resonance imaging (DW-MRI) data acquired by the Alzheimer’s Disease Neuroimaging Initiative (ADNI). A nonlinear dynamical module (Duffing oscillator) [27] is then assigned to each area, as the generator of its macroscopic electrical activity. A pathological state is defined as one in which all oscillators present high-amplitude theta-band frequencies. Conversely, in a healthy state, they oscillate with an alpha-band frequency. This designation of the pathological and healthy states seeks to match the slowing of electroencephalograms (EEG) induced by AD [28–34]. The control tasks consist of shifting the pathological activity of the nonlinear system to healthy activity, even though the damage the disease has caused to the patient is irreversible. The optimal signals for accomplishing this control objective are region and subject-specific, and obtained through SDRE. Brain regions can be ranked according to the energetic cost of performing the control task. These orders are nonlinearity-dependent. Interestingly, the regions associated with lower costs for the controller are those more topologically connected to the rest of the nodes in the network. These regions lie mostly in the limbic system or basal ganglia. On the other hand, subjects with compact networks (e.g., low average shortest path length, high clustering coefficient, high global efficiency,) can be controlled with more inputs to individual regions than the rest.

## Results

### Designing brain stimulation signals for AD: Linear vs nonlinear modeling approaches

We obtained anatomical connection density matrices, ***W***, from DW-MRI (*Methods*, *Data acquisition and processing*) for each of the 41 patients in ADNI (*Methods*, *Study participants*; S1 Table; S2 Table). We applied the SDRE optimal control framework (S1 Text) to a system of Duffing oscillators (*Methods*, *Dynamical systems)* representing the electrical activity of cortical and subcortical ‘pyramidal neuron’ populations coupled through the anatomical connection matrices. Specifically, we conceived a hypothetical experiment in which all the regions in the anatomical parcellation we used [35] can receive an input, but where each simulation has the input entering only one area.

**Fig 1:**
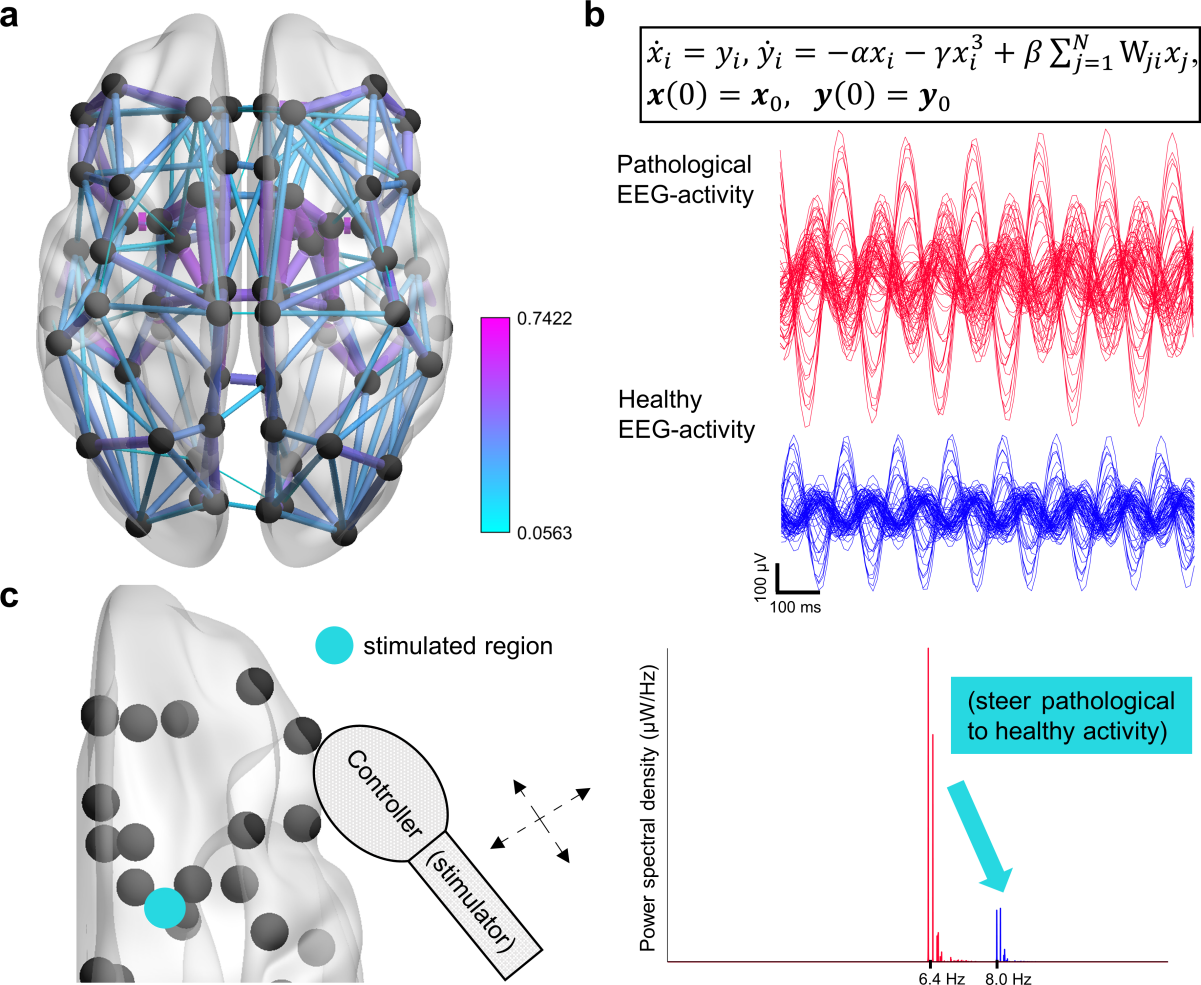
Optimal nonlinear network control of Alzheimer’s. **a**) Anatomical connection density matrices (***W***) for the interaction of 78 predefined brain regions were obtained for each of the patients in the study. The color code and size of the edges represent the weight of the connections. **b**) Duffing oscillators describe the activity in each brain region *i*, and are coupled through ***W***. The parameter *γ* characterizes the nonlinearity of the system. By tuning *α* and the initial conditions, ***z***_0_ = [***x***_0_,***y***_0_]^*T*^, ‘pathological EEG activity’ (high-amplitude theta-band oscillations, *f* ≈ 6.4 *Hz*) and ‘healthy EEG activity’ (low-amplitude alpha-band oscillations, *f* ≈ 8.0 *Hz*) are obtained. **c**) A hypothetical ‘controller’ is moved over all the regions. The controller applies the optimal (least energy-consuming) signal that steers the activity to the healthy state, and guarantees the shift of the EEG spectrum towards higher frequencies. Each stimulus depends on the region and patient receiving it through the dynamical system that is solved.

We calculated the minimal-energy input that converted pathological (theta-band) into healthy (alpha-band) activity, for every region and subject (*Methods*, *Control tasks)*. The rationale for this is the increase of power in the theta band (4.0–7.5 Hz) of the EEG spectrum, and the decrease of power in the alpha (8.0–12.5 Hz) and beta (13.0–32.0 Hz) bands in AD [28,29]. As reported by several studies, there is a correlation between cognitive impairment and the acuteness of EEG abnormalities [30]. Additionally, the use of cholinergic drugs (which can transiently shift the EEG spectra towards normality) was related to improved memory and attention performances in AD [32]. In another study, Babiloni et al. [33] found that theta sources of the EEG in parietal, occipital, temporal and limbic areas had higher magnitude in AD than in healthy controls, while alpha sources had lower magnitude in AD. Moreover, all the alpha sources showed positive correlations with the Mini Mental State Examination (MMSE) score for global cognitive level, suggesting the favorable impact of a shift towards elevated alpha activity. A different, posterior study also found positive correlations between alpha power and the patients’ MMSE scores [34]. Although a specific causal relationship between EEG rhythms and AD has not been established, brain stimulation that corrects EEG abnormalities will also positively affect the patient’s welfare by restoring cognitive performance, yet the disease is not cured. The persistence of the disease appears in our model through its parameters and the anatomical connection density matrices which is consistent with reports showing abnormalities in the graphs obtained from DW-MRI in AD [15,36,37]. The general scheme of our methodology is presented in Fig 1.

**Fig 2:**
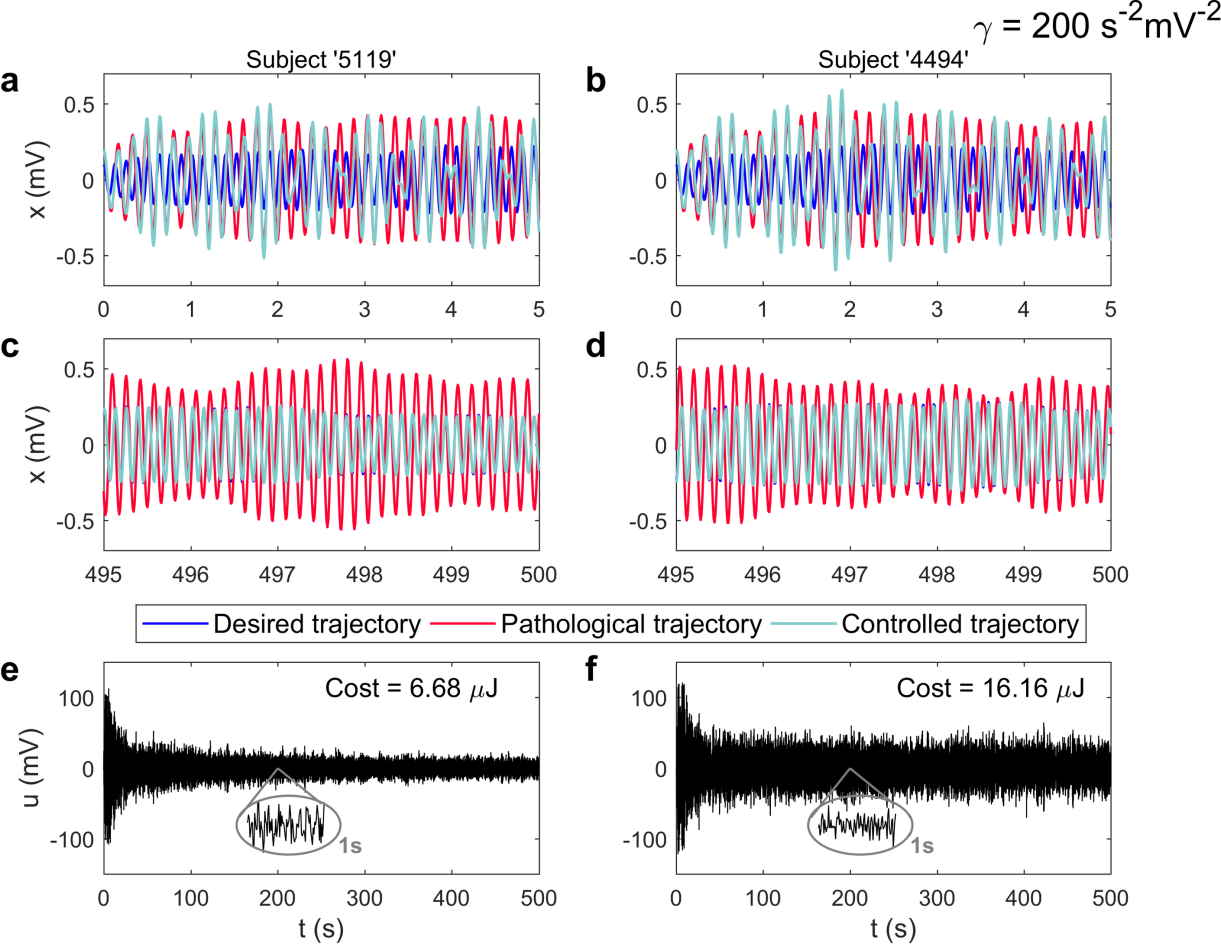
Controlling the Alzheimer’s pathological EEG activity (nonlinear case). **a**) Start of the simulations for the ADNI subject identified as ‘5119’. The evolution of the postsynaptic potential over one region is shown only. Others behave analogously. The desired trajectory corresponds to a ‘healthy’ low-amplitude alpha-band oscillation. The model can also produce ‘pathological’ high-amplitude theta-band oscillations. A control signal feeds the left pallidum for reverting the pathological activity. **c**) By the end of the simulation, the controlled trajectory almost identically matches the healthy state although it was created with the ‘pathological parameters’. This is the effect of the optimal control signal, shown in (**e**). Panels (**b**,**d**,**f**) present the same analysis for the subject identified as ‘4494’. Other inputs have the same effect over the impaired activity of subjects ‘5119’ and ‘4494’. However, when these signals are placed over the left pallidum, the energetic cost of the control task is minimum–inserted in (**e**) and (**f**). A one-second zoom-in window of the control signal at *t* = 200*s* is also inserted. The strength of the nonlinearity was set to *γ* = 200 *s*^−2^*mV*^−2^. See S1 Fig for equivalent results obtained over linear systems.

In contrast to conventional ideas on brain stimulation where identical signals are applied [4–8] regardless of subject-to-subject variability, we calculated a broad set of patient-specific signals that revert AD pathological activity, and studied their performance on the control tasks. Fig 2A, 2C and 2E show (respectively) the initial set-up of the temporal solutions of the nonlinear model, their behavior in the last five seconds of the simulated interval, and the optimal control signal, *u*(*t*), that hypothetically enters the left pallidum, in this example, and produces a successful control task. This corresponds to a specific subject in ADNI’s database. Fig 2B, 2D and 2F present the same analysis for a second subject. In both cases, the strength of the nonlinearity was *γ* = 200 *s*^−2^*mV*^−2^. This is a typical value among the strengths of the nonlinearity we tested (see S3 Table for a complete list of the parameters used throughout the study).

As seen from the temporal evolution of variable *x*, which represents the postsynaptic potential over one randomly chosen region in the model, the controlled trajectory almost identically matches the desired trajectory (low-amplitude alpha oscillation) by the end of the simulation (Fig 2C and 2D). Please, note the subtle differences in the signals the controller is set to deliver from one subject (Fig 2E) to the other (Fig 2F).

These dissimilarities are mostly due to the generation of subject-dependent minimal-energy signals. The magnitudes of the calculated optimal signals (-0.1–0.1 V, approximately) are around one order lower than the signals that are currently used in DBS for AD (3.0–3.5 V) [4,5]. The magnitude generally decreased with time although the signals possessed complicated shapes. The energetic cost of controlling the full network of oscillators was also computed. Roughly speaking, the energy was defined as the time-integral of the norm of the control input, *u*(*t*) (S1 Text). We found that low magnitude signals are associated with reduced costs (Fig 2E and 2F).

If the input was placed over a different region, the system might or might not be controllable. Several subject-dependent cases in which the optimal control framework failed to produce stimulation signals were obtained for nonlinear systems. S1 Fig shows equivalent results to those in Fig 2, although obtained over linear systems (*γ* = 0 *s*^−2^*mV*^−2^). No case of uncontrollable systems for any subject was found for signals entering the linear variant of the model though, which seems unrealistic to occur in any practical implementation. Additionally, the magnitude of the control signals obtained was generally lower for linear than for nonlinear systems. These results depend on the anatomical connection matrices and the dynamical model (coupled Duffing oscillators) we have used for the simulations.

### Selecting the target location for stimulation: Ranking the areas based on the cost of controlling the brain network

We collected the results of all simulations to construct a general picture of the power regions (nodes in the networks) have to control the AD system. Results for the simulations using the same strength of the nonlinearity, *γ*, were averaged across all the subjects in the study. An uncontrollable system is associated with an infinite energetic cost (whereas the inverse of the cost will be zero) since an infinite input signal would be required to correct pathological EEG activity in such a case. To overcome the presence of uncontrollable systems with proper visualization tools, we chose the inverse of the cost as the variable of interest for ranking the performance of the brain regions in our control tasks. Thus, a region with high inverse of the cost is associated with enhanced optimal control (the full network can be readily controlled with an input entering such region).

**Fig 3:**
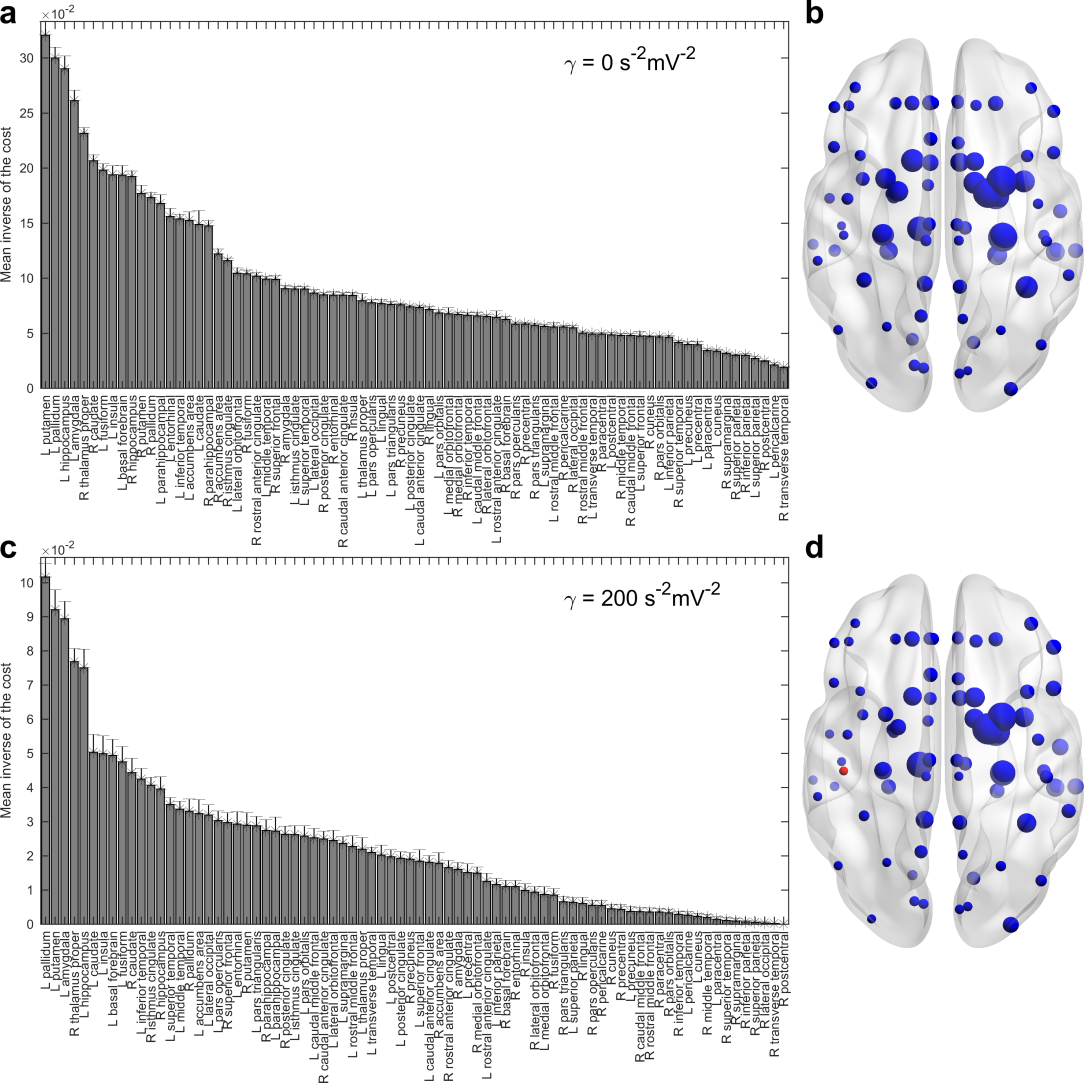
Ranking brain regions according to the mean inverse of the cost of controlling the network. **a**) Order corresponding to the linear case. Given is the mean ± SEM of N = 41 subjects. Inputs entering regions in the leftmost part of the order control Alzheimer’s activity at a lowest cost. **b**) Graphical representation with the approximated location of the brain regions. The size of the spheres is directly proportional to the mean values in panel (**a**). Panels (**c**,**d**) are analogous to (**a**,**b**) except that the strength of the nonlinearity has been set to *γ* = 200 *s*^−2^*mV*^−2^ and a new ranking is obtained. The red sphere represents the right postcentral gyrus, which yielded uncontrollable nonlinear systems for all the subjects in the sample.

Fig 3A shows the brain areas’ ranking for the limit case of a linear system (mean inverse of the cost **±** standard error of the mean). Top-ranked areas appear in the leftmost part of the panel. Fig 3B contains a graphical visualization of the brain sites where they are approximately located. The size of the spheres is directly proportional to the inverse of the cost. We found that several of the top-ranked regions are spatially close, with predominance over the left hemisphere. New rankings were obtained when the nonlinearities increased (see Fig 3C and 3D). As the magnitudes of the costs generally grow with the strength of the nonlinearity, the upper limit of the vertical axis in Fig 3C, representing the maximum mean inverse of the cost registered for *γ* = 200 *s*^−2^*mV*^−2^, is smaller than the corresponding one in Fig 3A.

**Fig 4:**
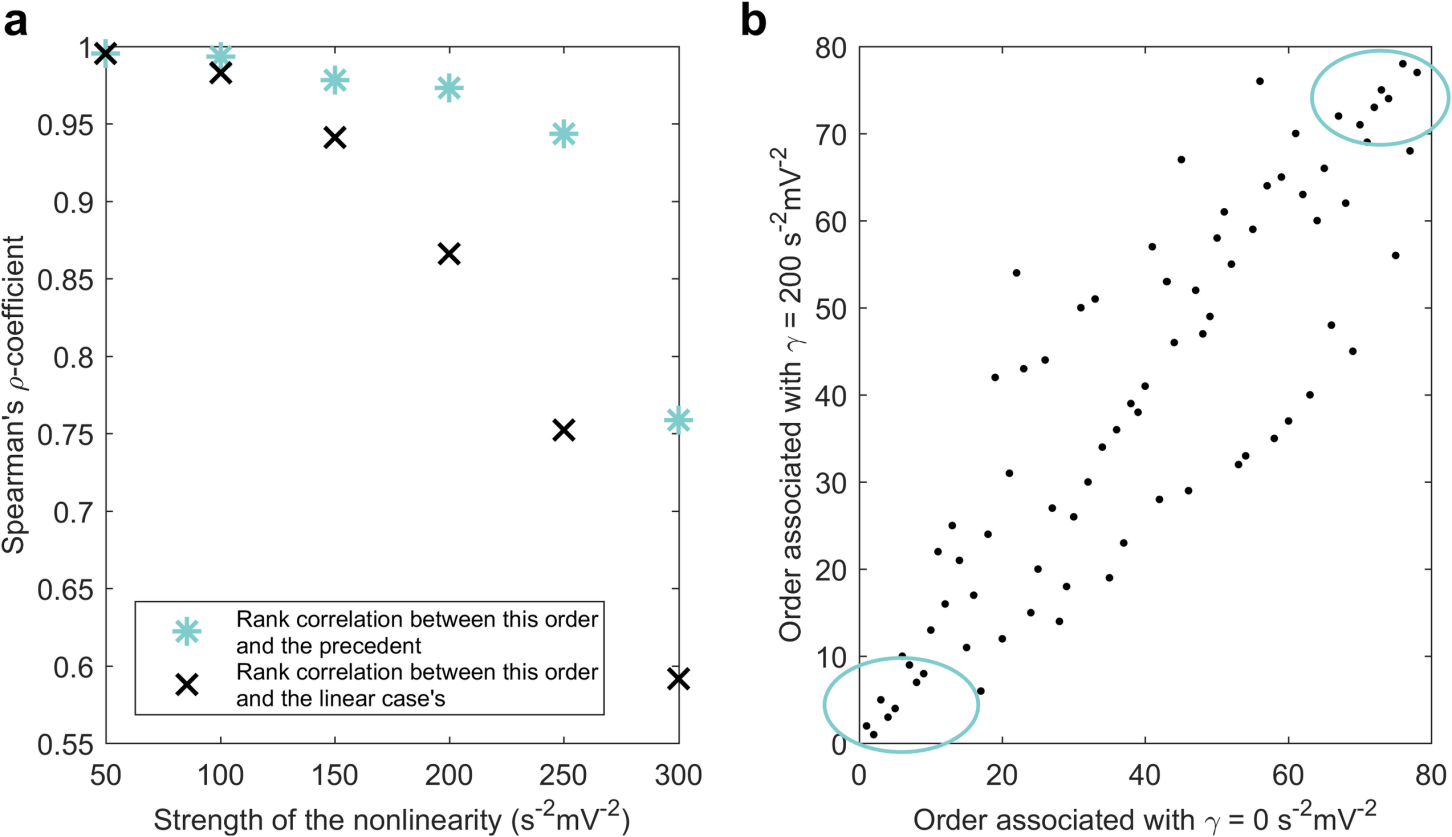
Nonlinearity-related changes to the average brain regions’ ranking. **a**) Rank correlations between the orders corresponding to different nonlinearities (paired t-test: large-sample approximation, P < 0.001 in all the cases, N = 78 regions). As the nonlinearity increases, the Spearman’s rho coefficients for the correlation between a ranking and both, the order corresponding to the previous nonlinearity and to the linear case, decrease. **b**) The rankings for the nonlinearities *γ* = 0 *s*^−2^*mV*^−2^ and *γ* = 200 *s*^−2^*mV*^−2^ are compared. These orders are similar in their top and bottom-most parts (inserted ellipses) and dissimilar in between.

Additionally, we assessed the relationship between the rankings of the regions resulting from controlling systems with different nonlinearities. The statistical dependence between the rankings associated with the nonlinearities was measured in terms of Spearman correlation (Pearson correlation between the rankings). The Spearman’s rank correlation coefficient (Spearman’s rho) between the linear system’s order and the corresponding to a nonlinear system with *γ* = 100 *s*^−2^*mV*^−2^ was *ρ* = 0.98 (*p* < 0.001). It decreased to *ρ* = 0.87 (*p* < 0.001) when the nonlinearity was increased to *γ* = 200 *s*^−2^*mV*^−2^ and further down to *ρ* = 0.59 (*p* < 0.001) for *γ* = 300 *s*^−2^*mV*^−2^. The orders corresponding to two consecutive nonlinearities also differ more (Fig 4A).

These ‘expected’ rankings, obtained from looking at the average inverse of the cost only, had similarities in their top and bottom-most components (Fig 4B), suggesting a global privileged/disadvantageous position of some areas in the brain network that transcends the effects of the nonlinearities. We consider it important to note that individual cases of uncontrollable systems were ubiquitously reported when nonlinearities were considered. Only the individual calculation of the minimal-energy control signals, instead of an analysis over the main values as performed in this section, can conduce to a trustable subject-specific selection of stimulation targets.

Nevertheless, it is interesting to note how regions on the top of the mean control order (Fig 3A and 3C) belonged to a clearly defined group with prevalence in the left hemisphere: the left pallidum, left putamen, left amygdala, left hippocampus, right thalamus proper, left insula, left basal forebrain, left fusiform and the caudate nuclei. Overall, these high-ranking regions belong to the limbic system and the basal ganglia. On the other hand, the worst-ranked areas included the right postcentral gyrus, both paracentral lobule, right inferior and superior parietal lobules, left cuneus and the right temporal lobe, which can all be classified as temporoparietal regions. Temporal and parietal cortical areas are affected in AD early in the disease course [4].

### Network topology helps to select stimulation candidates

Identifying the most suitable candidates for a successful brain stimulation treatment remains a challenge. Even subjects suffering from the same condition are intrinsically different due to genetic and environmental factors [38]. Here, we aimed to create a gold standard for selecting both stimulation sites and individuals most likely to benefit from stimulation therapy based on the subject’s anatomical networks estimated from DW-MRI data. We looked at the relationship between the results of the control tasks and the topological characteristics of the networks, ***W***′*s*, over which they are performed (S2 Text).

**Fig 5:**
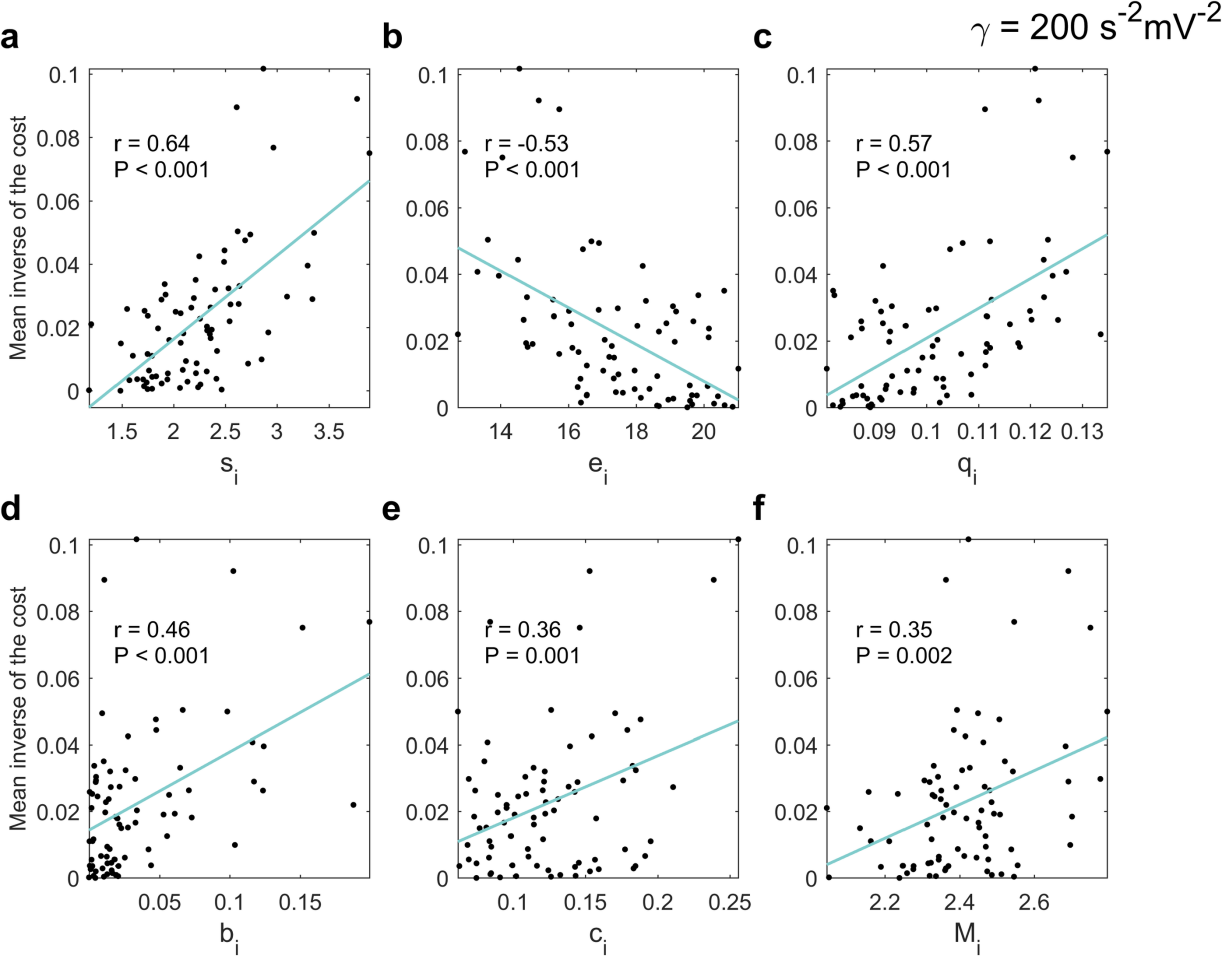
The effect of the local topological measures on the performance of the controllers (nonlinear case). Relationship between the mean inverse of the cost across the sample and the mean node strength (**a**) (linear regression: F(1,76) = 55.95, P < 0.001), eccentricity (**b**) (linear regression: F(1,76) = 29.61, P < 0.001), closeness centrality (**c**) (linear regression: F(1,76) = 36.94, P < 0.001), betweenness centrality (**d**) (linear regression: F(1,76) = 20.90, P < 0.001), clustering coefficient (**e**) (linear regression: F(1,76) = 11.36, P = 0.001) and communicability (**f**) (linear regression: F(1,76) = 10.87, P = 0.002); N = 78 regions, in all cases. The Pearson correlation coefficients, *r*, are inserted. The strength of the nonlinearity was set to *γ* = 200 *s*^−2^*mV*^−2^. See S2 Fig for equivalent results obtained over linear systems.

To gain further insight into the regions offering the best optimal control perspectives, we studied the relationship between the mean regional inverse of the costs and average local measures. The chosen topological measures were node strength, *s*_*i*_; eccentricity, *e*_*i*_; closeness centrality, *q*_*i*_; betweenness centrality, *b*_*i*_; clustering coefficient, *c*_*i*_ and communicability, *M*_*i*_. In Fig 5A–5F we show the above-mentioned dependences. These topological quantities classify the degree of ‘importance’ a node has in the network. For example, a low eccentricity denotes short paths from a node to the rest of the network, which is also interpreted as a high closeness centrality, whereas communicability counts direct and indirect paths of all lengths between two nodes. The strength accounts for both, the number of connections a node has and the value of the connection weights. On the other hand, high values of betweenness centrality relate to nodes that act as bridges in the network and a high clustering coefficient means tendency to form triangles, or cluster together.

We found significant correlations between the mean inverse of the cost of controlling the brain network from a region and all the local measures (*s*_*i*_, *e*_*i*_, *q*_*i*_, *b*_*i*_, *c*_*i*_ and *M*_*i*_). The only decreasing relationship found was with the eccentricity, *e*_*i*_, meaning that regions with a small shortest path length might constitute the more suitable targets for controlling the network. On the other hand, nodes with high *s*_*i*_, *q*_*i*_, *b*_*i*_, *c*_*i*_ and *M*_*i*_ were associated with high mean inverse of the cost. Analyzing the strength of the correlations revealed an interesting pattern: the three correlation coefficients appearing on the top row of Fig 5 –for quantities strictly related to direct connections–were considerably higher than those on the bottom which relate to measures for quantifying relay nodes, segregation levels and indirect paths, respectively. This suggests that direct links (high weights, small shortest paths) between nodes are what makes a stimulus fully propagate over a network to reach the control objective at a low energetic cost.

**Fig 6:**
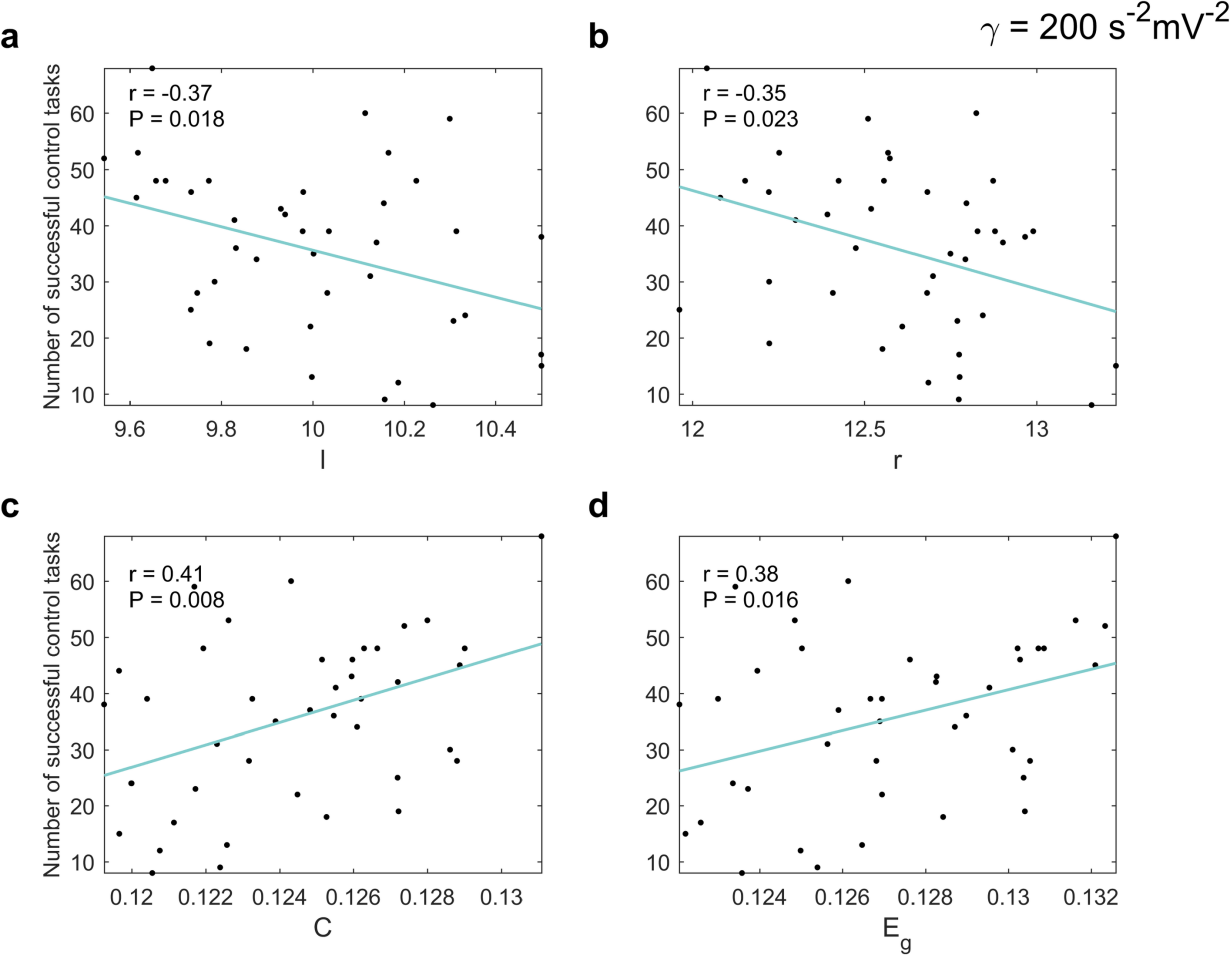
The effect of the global topological measures on the success of the control tasks. Relationship between the number of successful control tasks per subject–the maximum possible value being 78– and the characteristic path length (**a**) (linear regression: F(1,39) = 6.08, P = 0.018), radius (**b**) (linear regression: F(1,39) = 5.60, P = 0.023), average clustering coefficient (**c**) (linear regression: F(1,39) = 7.77, P = 0.008), and global efficiency (**d**) (linear regression: F(1,39) = 6.39, P = 0.016); N = 41 subjects, in all cases. The Pearson correlation coefficients, *r*, are inserted. The strength of the nonlinearity was set to *γ* = 200 *s*^−2^*mV*^−2^. There are no linear systems-equivalent results as all the stimuli yielded controllable systems in that case.

As previously expressed, a set of inputs entering certain specific regions for each subject failed to convert theta activity into alpha activity. The number of successful signals thus provides a good estimate of how responsive the patients would be to the tentative treatment herein modeled. Therefore, we studied the relationship between the number of inputs resulting in controlling the systems and global measures of the subjects’ anatomical network. This is shown in Fig 6A–6D (characteristic path length, *l*; radius, *r*; average clustering coefficient, *C*, and global efficiency, *E*_*g*_, in this order). We found that subjects with small average shortest path length (*l*) of their anatomical networks were controlled by more inputs–in other words, from a high number of regions. In the same way, the lower the radius was, the more inputs were efficient in the control tasks. More clustered networks yielded the same result. Small average distance between the nodes in the network and high clustering coefficient are attributes associated with the small-worldness property [39], a concept that relates the fast spread of stimuli to the existence of ‘shortcuts’ in a network. Finally, the number of areas from which the AD brain could be controlled per subject was also proportionally related to the global efficiency, a measure that reflects how efficiently information can be exchanged over the network. We did not obtain any significant correlation between the number of controllable dynamical systems and the diameter of the networks.

What is presented here stands for all the strengths of the nonlinearity we tested. All the results in this section corresponded to *γ* = 200 *s*^−2^*mV*^−2^. However, the magnitudes of the correlation coefficients were higher as the strength of the nonlinearity decreased. The same analysis presented in Fig 5 can be found in S2 Fig for the linear systems.

## Discussion

The goal of brain stimulation is to exogenously control (i.e., manipulate) the brain’s activity so that it follows a desired pattern associated with a healthy state [11]. The specific characteristics of the signals in brain stimulation experiments/therapies are usually overlooked. Square pulses are translated from the treatment of one condition to the other (e.g., from Parkinson’s to Alzheimer’s) [4,5], sometimes tuned in an exploratory way, and applied identically to every subject without considering individual differences. In a world where medicine is constantly becoming more personalized, treatments which are designed using broad statistical measures and account poorly for interpatient variability are inefficient [38]. Current approaches to modeling brain stimulation present major shortcomings (see the recent review by Bassett et al. [13], for example) and importantly, nonlinearities are known to characterize the brain’s dynamical behavior and should not be excluded from any realistic modeling.

In this work, we introduced a framework for calculating the optimal signals and most suitable regional targets in the brain for controlling AD activity, catered to individual subjects. Unlike other studies [9,11,12], ours considers the existence of nonlinearities in the modeling of brain dynamics by extending the use of the so-called state-dependent Riccati equation control to biological, high-dimensional systems. The calculation of the optimal signals that can propagate over the network and set its temporal dynamics to a desired control state also provides insights into the way neural systems control themselves. If a network node associates with low cost for exogenously controlling the neural system, then that same element must have certain advantaged position for the self-regulation processes occurring there. Importantly, our methodology is not restricted to AD. Any other clinical condition characterized by abnormalities in brain dynamics (and with existing meaningful neuroimaging data for using in the modeling process) could be addressed similarly with: 1) a model for the dynamics is assumed, 2) the model is set to produce pathological and healthy activity, and 3) brain stimulation signals that revert pathological activity at the lowest possible energetic cost are found through SDRE.

We modeled a stimulation-therapy for AD based on the correction of the EEG spectrum towards higher frequencies. As such, we looked for inputs (control signals) to individual areas of the brain that revert pathological activity at the lowest possible energetic cost. Among all the possible signals that were obtained for each subject, the one producing the fastest, least energy-consuming response, can be administered in a brain stimulation procedure. On the other hand, the SDRE-framework allows us to naturally identify those brain regions into which stimulation would not contribute to achieving the control objective (by using the definition of uncontrollable nonlinear system). The right postcentral gyrus, for example, yielded uncontrollable systems for all the subjects in the sample.

We also studied the dependence of the optimal control tasks on the anatomical networks conditioning the dynamics. In essence, we found a strong relationship between the success of the control tasks and the topological features of the anatomical connection density matrices that served as scaffold for the interaction of the cortical and subcortical ‘pyramidal neuron’ populations in the model. Overall, the significant correlations existing between the mean inverse of the cost and the local topological measures suggest that nodes with high connectivity associate with low cost of controlling the full network of oscillators. Our results agree with previous findings that stimulation to strongly connected nodes in brain networks produces low-energy transitions [11,12]. For each subject, we found that the better connected a network is–namely, a network having low average shortest path length, high clustering coefficient and/or high global efficiency–, the more inputs to individual nodes success on the control task and make the system evolve to the predetermined healthy state (subjects having ‘a better-connected network’ are the optimal candidates for AD’s effects-reverting protocols). However, these indications relating optimal nonlinear control and network measures are only an approximation (based on average values), and we recommend the calculation of the optimal signals and targets for each subject to undergo our proposed brain stimulation for AD.

The inclusion of nonlinearities in our model causes several control tasks to fail for each subject, a fact that, to the best of our knowledge, has not been reported for linear brain dynamics. However, we do not expect that inputs to every neuronal conglomerate in real stimulation experiments are able to steer the (AD) brain to the desired state, given its complexity and nonlinear character [18–20]. The order in which areas were ranked according to the energy used for controlling the network, changed with the strength of the nonlinearity, *γ*. Interestingly, as *γ* increased, the linear dependence of the expected cost of controlling from a region on its topological characteristics was less obvious (see Fig 5 and S2 Fig)–the higher the *γ* is, the less the systems look like sets of linear (harmonic) oscillators coupled through the anatomical connection density matrices. This likely denotes competition between the effects of the nonlinearity and the structure of the network for the dynamical interaction, and warrants further investigation. Overall, our findings reveal the importance of using nonlinear realistic modeling to better understand brain stimulation and its accurate design.

When ranking the regions in the brain according to the average cost of controlling the network with a single stimulus, we found that the lowest energetic cost was associated with limbic and basal ganglia areas, or strongly connected to them, such as the thalamus. The role of these areas in motor control, learning, memory and relay of information [40,41] engages them in a wide number of connections, and consequently (see Fig 5), makes them highly desirable targets for stimulation. The globus pallidi sends basal ganglia information to the thalamus which projects back to the cortex [41]. Specifically, the left pallidum–at the top of the nonlinear systems ranking–has been previously identified as having the least overall multifactorial damage by AD [1]. The caudate nuclei and putamen receive and process cortical and thalamic information which is later transmitted to the globus pallidi [41]. On the other hand, the large-scale brain network topology seems to be organized to concentrate information flow in the hippocampal formation [42], structure with a key role in memory processing [43], and also among those associated with better optimal nonlinear network control in this work. Finally, the amygdala has a broad pattern of anatomical connections, especially with other subcortical structures [44], making it another of the top targets for achieving successful control tasks.

The bulk of the poorly-ranked areas comprised temporal and parietal association cortices and sensory and motor cortices structures. Interestingly, most of these bottom-ranking areas are in the right hemisphere. Some experimental evidence supports this finding, such as reports of increased vascular and AD burden (amyloid-β and tau deposition) in the right hemisphere, compared to the left [45]. Additionally, in one of the studies that inspired this work [4], no downstream evoked response in the right hemisphere was recorded for one patient out of six. They performed DBS of the fornix, an axonal bundle that acts as a major output and input tract for the hippocampus and the temporal lobe. The absence of a right-sided response in some subjects while indirectly stimulating several regions simultaneously, along with the recorded worsening of AD in the right hemisphere may explain the low performance of right hemisphere controllers in our work.

Most top-ranked regions were subcortical structures (e.g., pallidum, amygdala, thalamus proper, hippocampus). However, other similarly-ranked areas, such as the insula, are cortical. Current brain stimulation techniques differ in reach, design and degree of invasiveness. In therapeutic practice, either one (subcortical structures) or the other (cortical structures) are targeted [6–8]. In a recent work, noninvasive DBS of the hippocampus in living mice was achieved by Grossman et al. while applying alternating high frequency currents at slightly different frequencies over the scalp [46]. Although the pattern of currents used by Grossman et al. (sinusoidal-like) is simpler than the ones we have obtained (Fig 2), their work shows the possibility of stimulating neuronal sets at any depth by using superficial devices. As such, the most suitable regional target for each patient (either subcortical or cortical) could be reached by using a single device. In a previous study, Terney et al. introduced current stimulation by high-frequency noisy signals [47] with positive results. The temporal profiles of the signals administered in that study somehow resembles the ones we obtained, although theirs have higher frequency, amplitude and seemingly noisier components. Together, these works indicate the feasibility of our proposal in terms of designing a device that delivers tailored signals to any location in the brain. We predict an eventual merging of our theoretical approach with cutting-edge stimulation technology like the ones proposed in the referred studies.

Another issue regarding the future development of optimal nonlinear network control of AD is the possibility of the spilling of stimulation to adjacent nuclei [48,49] as we propose to target single localized regions. Nonetheless, the lack of focality of brain stimulation techniques might be an advantage for their clinical application [49]. Several of the regions from which the desired trajectory was achieved at low energetic cost in our model have physical proximity (see Fig 3B and 3D), and could be reached in a target-specific experiment [48]. We hypothesize that simultaneous stimulation of different structures would produce faster optimal control of the pathological activity. Spilling and the intentional stimulation of selected structures with different signals are other modeling possibilities we will assess in a separate work.

On the other hand, the recently-introduced adaptive deep brain stimulation (aDBS) is gaining support for replacing the conventional constant-parameters brain stimulation in the treatment of Parkinson’s [21–24]. aDBS uses the subthalamic local field potential (LFP) activity recorded directly from the DBS electrode itself as a feedback for tuning the stimulation signal in real time. The level of beta frequency band oscillations in the LFP correlates with motor impairment, in the presence or absence of therapeutic interventions [21]. A brain–computer interface system uses this biomarker to control when the stimulation is applied. Thus, aDBS is a closed-loop technology [23]. Such procedure delivers less energy to the patient (with fewer side effects) and is clinically superior to standard continuous DBS, according to the results reported in several studies [21,22]. Our framework, designed without knowledge of the existence of aDBS, aims to obtain stimulation protocols that are also assembled over the analysis of a feedback signal related to the patient’s clinical condition (see equation (S1.5)). The successful application of aDBS supports the use of closed-loop approaches for stimulation, such as the one we have introduced.

Finally, we would like to point out the pioneering nature of our work and list its methodological limitations in what follows. Further work is to be done in solving those before proceeding to demonstrate the efficacy of our approach in actual brain stimulation experiments for AD (either in animal models or human subjects). The main issue that needs to be addressed is replacing the parameters in our model with real values estimated from the analysis of a patient’s electrical activity. The selection of the dynamical model used in this work was based on its relative mathematical simplicity (it offers the possibility of assessing both linear and nonlinear cases by switching a single parameter) while still resembling broadly used electro-physiologically-inspired neural mass models [18,50]. Several techniques for estimating its parameters are available, with outstanding results emerging from the use of the innovation method based on local linearization filters [51,52].

However, the estimation of the effective connectivities [53] mediating the interaction between neuronal populations (78 × 78 values in our case) might constitute a computationally costly problem. This is why, inspired by previous approaches [10–12], we focused on the ‘structural side’ of connectivity for optimally controlling the AD’s brain. Both functional and effective connectivity correlate to structural connectivity [54,55]. In this work we assumed that the strength of the structural connectivity was proportional to a measure derived from diffusion MRI tractography–the anatomical connection densities [56]. Nevertheless, there is a consensus on the limited performance of tracking algorithms and anatomically-imposed difficulties that suggests prudence in making such assumption [57,58]. An inherent limitation of DW-MRI is its inability to detect the direction of nervous fibers [56], which extends to all current neuroimaging methods [58]. However, a substantial proportion of reciprocal connections has been identified [59], justifying the ubiquitous use of undirected anatomical networks. Additionally, variability across DW-MRI studies and methods [57], constitutes a major issue to deal with for achieving generalization. A finer partitioning of the brain into regions could also result in more localized targets for clinical stimulation.

The controllers we designed have lower magnitude–with lower associated energy deposition–than what has been identified as the safety threshold [4,5] in DBS for AD (3.0–3.5 V) and are still (computationally) successful in the reversion of pathological activity. Whereas the shape of our signals is in-between the low-frequency sinusoidal-like inputs that Grossman et al. [46] applied and the high-frequency random noise stimulation performed by Terney et al. [47] with no side effects, further research must be carried on to address safety concerns. Our controllers might have a negative outcome–as any other current delivered to the brain tissue. Few adverse effects are generally reported when weak electrical currents are administered to the scalp (e.g. through tDCS) [3]. High-frequency invasive stimulation (DBS) causes stronger side effects overall [60]. Thus, the application of our signals should be preceded by a complete *in vivo* assessment of their impact on the tissue.

Another of the limitations of our model in its current state is assuming that all nodes present the same time constants (S3 Table), yielding to approximately equal oscillation frequencies (~6.4 Hz for the ‘pathological state’, 8.0 Hz for the ‘healthy state’). Variability was the result of the interaction with other regions through the anatomical networks only. Apart from the strength of the nonlinearity, the existing differences in the nodes’ natural frequencies [61] can also affect the outcomes of the control tasks. To address this specific issue, we performed an exploratory analysis over one of the subjects in the database, in which each node was assigned a random natural frequency inside the theta and alpha bands. A more detailed discussion can be found in S3 Text and S3 Fig. In short, the variation did affect the ability of the controllers to revert the disease consequences, by both increasing the energetic cost and producing more tasks to fail. However, randomly generated frequencies also lack physiological meaning. As previously stated, no definitive control strategy can be delineated until the calculations are performed over models that include as much realistic information as possible, i.e., estimating the actual oscillation frequencies from the subject’s electrical activity.

Even with limitations in the modeling at this very primary stage and the need for experimental validation, the results herein reported constitute a progress, and overall, this work might represent a change to the methodology for addressing the control principles of the brain. Our future research intends to use multimodal data to overcome the above-stated imperfections. Our ultimate goal is to design controllers for efficiently and realistically reverting pathological states of each patient’s brain activity.

## Methods

### Ethics statement

The study was conducted according to Good Clinical Practice guidelines, the Declaration of Helsinki Principles, US 21CFR Part 50—Protection of Human Subjects, and Part 56—Institutional Review Boards, and pursuant to state and federal HIPAA regulations (adni.loni.usc.edu). Study subjects and/or authorized representatives gave written informed consent at the time of enrollment for sample collection and completed questionnaires approved by each participating sites Institutional Review Board. The authors obtained approval from the ADNI Data Sharing and Publications Committee for data use and publication, see documents http://adni.loni.usc.edu/wp-content/uploads/how_to_apply/ADNI_Data_Use_Agreement.pdf and http://adni.loni.usc.edu/wp-content/uploads/how_to_apply/ADNI_Manuscript_Citations.pdf, respectively.

### Study participants

This study used 41 individual baseline data from ADNI. Structural magnetic resonance images (MRI) and diffusion weighted MRI (DW-MRI) were acquired for each of the ADNI subjects included in the study. We used the individual clinical diagnoses assigned by the ADNI experts, which were based on multiple clinical evaluations. The 41 subjects were diagnosed as Alzheimer’s patients. The ADNI was launched in 2003 as a public-private partnership, led by Principal Investigator Michael W. Weiner, MD. The primary goal of ADNI has been to test whether serial magnetic resonance imaging, positron emission tomography, other biological markers, and clinical and neuropsychological assessment can be combined to measure the progression of mild cognitive impairment and early AD.

See S1 Table for a complete list of the included ADNI subjects and S2 Table for demographic characteristics.

### Data acquisition and processing

#### Structural MRI

Brain structural T1-weighted 3D images were acquired for all subjects. For a detailed description of acquisition details, see http://adni.loni.usc.edu/methods/documents/mri-protocols/. All images underwent non-uniformity correction using the N3 algorithm [62]. Next, they were segmented into grey matter, white matter and cerebrospinal fluid (CSF) probabilistic maps, using SPM12 (www.fil.ion.ucl.ac.uk/spm). Grey matter segmentations were standardized to MNI space [63] using the DARTEL tool [64]. Each map was modulated to preserve the total amount of signal/tissue. Mean grey matter density and determinant of the Jacobian (DJ) [64] values were calculated for 78 regions covering all the brain’s grey matter [35].

#### Diffusion weighted MRI

High angular resolution diffusion imaging (HARDI) data was acquired for 41 subjects. For each diffusion scan, 46 separate images were acquired, including 5 *b*_0_ images (no diffusion sensitization) and 41 diffusion-weighted images (*b* = 1000 *s*/*mm*^2^). Other acquisition parameters were: 256 × 256 matrix, voxel size: 2.7 × 2.7 × 2.7 *mm*^3^, *TR* = 9000 *ms*, 52 contiguous axial slices, and scan time, 9 *min*. ADNI aligned all raw volumes to the average *b*_0_ image, corrected head motion and eddy current distortion.

#### Anatomical networks

The T1-weighted 3D anatomical images were registered to the *b*_0_ images using a normalized mutual information method [65]. Probabilistic axonal connectivity values between each brain voxel and the surface of each considered gray matter region (voxel-region connectivity) were estimated using a fully automated fiber tractography algorithm [56] and the intravoxel fiber distributions (ODFs) of 41 diseased subjects from ADNI. ODF reconstructions were based on Spherical Deconvolution [66]. A maximum of 500 *mm* trace length and a curvature threshold of ±90° were imposed as tracking parameters. Based on the resulting voxel-region connectivity maps, the individual region-region anatomical connection density matrices [56], ***W***, were calculated. For any subject and pair of regions *i* and *j*, the *W*_*ji*_ measure (0 ≤ *W*_*ji*_ ≤ 1,*W*_*ji*_ = *W*_*ij*_) reflects the fraction of the region’s surface involved in the axonal connection with respect to the total surface of both regions. A network backbone, containing the dominant connections in the average network, was computed using a minimum-spanning-tree based algorithm [58] and used as a mask for all the subjects’ connection maps.

### Dynamical systems

We choose a mathematical model that balances simplicity and physiological reliability. This consists of a set of Duffing oscillators [27], linearly coupled through the anatomical connection density matrices, **W** ∈ ℝ^*N*×*N*^. Here, *N* is the number of nodes in the network (*N* = 78). The state variables ***x*** are interpreted as excitatory postsynaptic potentials in a neural mass formulation [50,67] (units: *mV*). In the low activity limit, the sigmoid activation function in these models can be replaced by a third-order approximation [68]. Then, in the model we use, the dynamics in area *i* is described by:

$${\dot{x}}_{i}=y_{i}$$

$${\dot{y}}_{i}={-\alpha x}_{i}-\gamma x_{i}^{3}+\beta \sum_{j=1}^{N}W_{ji}x_{j},$$ (1)

$$x(0)=x_{0},\;y(0)=y_{0}$$

where ***z*** = [***x***,***y***]^*T*^ ∈ ℝ^*n*^ (*n* = 2*N*) is the state vector and *β* is the strength of the coupling. The parameter *γ* is the strength of the nonlinearity. The limit case of a linear system can be readily achieved by making *γ* = 0. Additionally, the amplitude of the solution grows with the initial condition, ***x***_0_ and the frequency, with *α* [27]. Any desired feature of the spectrum related to the system in (1) (healthy or pathological activity in AD) can be simulated by tuning these parameters. We distinguish two different solutions, ***z***, of (1) based on the values of the parameter *α* (and the initial conditions, ***z***_0_ = [***x***_0_, ***y***_0_]^*T*^). Let us call these solutions ***z***_*p*_, if *α* = *α*_*p*_ (produces high-amplitude theta-band oscillations), and ***z***_*h*_, if *α* = *α*_*h*_ (produces low-amplitude alpha-band oscillations). Throughout this paper we use subscripts ‘*p*’ and ‘*h*’ to denote pathological and healthy states, respectively. The specific set of parameters used in this work appears in S3 Table.

### Control tasks

The control task consists of steering the nonlinear system that results from making *α* = *α*_*p*_ in (1) to the one obtained when *α* = *α*_*h*_ by applying an input that enters only one node in the network. Thus, we search for stimuli that make the difference between ***z***_*p*_ and ***z***_*h*_, ***e*** =***z***_*p*_ − ***z***_*h*_, as small as possible [69]. Those control signals revert the increase in the theta-band power registered in AD and steer it to a ‘healthy activity’, even though the underlying pathological system–given in the model by the parameter *α*_*p*_ and the affected anatomical networks, ***W***– is unchanged. There is a correlation between abnormalities in EEG spectral measures and severity of dementia; drug-induced transient restoration of EEG normality is related to improved attention and memory performances [30,32]. We include these findings as key elements for modeling optimal brain stimulation for AD.

The dynamical equations for ***e*** can be written generically, under certain conditions [26], as:

$$\dot{e}(t)=A(e)e(t)+Bu(t),\;e(0)=e_{0},$$ (2)

where *u*(*t*) symbolizes the external (control) signal and ***B*** is a vector whose components are only different from zero at the entry corresponding to the *y*-variable in the region receiving the input. By changing the position of this non-zero element, we cover all possible ‘stimulations’ to single regions. New, different systems are also generated each time the connectivity matrices, ***W***, are changed. The optimal feedback control signal *u*(*t*) is obtained from solving the so-called state-dependent Riccati equation (SDRE) [25,26,69,70]. This input exists if system (2) is observable and controllable. Observability is guaranteed with a careful though simple selection of weights in the SDRE framework [16]. The state-dependent linearization in (2) yields a controllable system in a region ℧ ∈ ℝ^*n*^ if the matrix [***B***|***A***(***e***)***B***|…|***A***^*n*−1^(***e***)***B***] has rank *n* for every ***e*** ∈ ℧ [26,69]. See S1 Text for more details on the SDRE theory and the explicit form of matrix ***A***.

### Numerical methods and data analysis

All the simulations and analysis in this work were implemented and performed within MATLAB R2017a (The MathWorks Inc., Natick, MA, USA). Visualization of the results was partially performed by means of BrainNet Viewer [71]. The systems in (2) were iteratively solved using a Local Linearization scheme, which is known to be stable and preserves nonlinearities [72,73]. The evaluation of (2) at each iteration conduced to locally linear systems. The solution to the SDRE was obtained by using MATLAB’s ‘lqr’ function. In practice, the controllability condition is checked while the numerical integration of the system is performed. Thus, the assessment of the controllability condition was also pointwise-managed through ‘lqr’, as it returns an error for uncontrollable systems.

We studied the relationship between the mean cost of controlling the entire network from a region and the total number of controllable systems (regions from which the input propagates to the whole network and reverts the pathological activity) per subject, and local and global network topological measures, respectively. The topological quantities we considered were: node strength, eccentricity, closeness centrality, betweenness centrality, clustering coefficient, characteristic path length, radius, diameter, average clustering coefficient and global efficiency. The Brain Connectivity Toolbox [58] was used for calculating the above-mentioned indices. A related measure, the communicability [74], that accounts not only for the shortest path lengths communicating two nodes in a network, but also for indirect connections that permit information to travel, was computed as well. The quantities herein used are weighted [14,75] since the anatomical connection densities can take any value from 0 to 1. Definitions and further information can be found in S2 Text.

## Supporting information

S1 Fig Controlling the Alzheimer’s pathological EEG activity (linear case). **a**) Start of the simulations for the ADNI subject identified as ‘5119’. The evolution of the postsynaptic potential over one region is shown only. Others behave analogously. The desired trajectory corresponds to a ‘healthy’ low-amplitude alpha-band oscillation. The model can also produce ‘pathological’ high-amplitude theta-band oscillations. A control signal feeds the left pallidum for reverting the pathological activity. **c**) By the end of the simulation, the controlled trajectory almost identically matches the healthy state although it was created with the ‘pathological parameters’. This is the effect of the optimal control signal, shown in (**e**). Panels (**b**,**d**,**f**) present the same analysis for the subject identified as ‘4494’. The energetic cost of the control task is inserted in (**e**) and (**f**). A one-second zoom-in window of the control signal at *t* = 200*s* is also inserted. The strength of the nonlinearity was set to γ = 0 *s*^−2^*mV*^−2^. This figure is the linear systems-equivalent to Fig 2 in the main text.
(TIF)

S2 Fig The effect of the local topological measures on the performance of the controllers (linear case). Relationship between the mean inverse of the cost across the sample and the mean node strength (**a**) (linear regression: F(1,76) = 105.72, P < 0.001), eccentricity (**b**) (linear regression: F(1,76) = 46.58, P < 0.001), closeness centrality (**c**) (linear regression: F(1,76) = 55.70, P < 0.001), betweenness centrality (**d**) (linear regression: F(1,76) = 25.20, P < 0.001), clustering coefficient (**e**) (linear regression: F(1,76) = 18.70, P < 0.001) and communicability (**f**) (linear regression: F(1,76) = 18.26, P < 0.001); N = 78 regions, in all cases. The Pearson correlation coefficients, *r*, are inserted. The strength of the nonlinearity was set to *γ* = 0 *s*^−2^*mV*^−2^. This figure is the linear systems-equivalent to Fig 5 in the main text.
(TIF)

S3 Fig The effect of the variability in the regions’ natural frequencies on the performance of the controllers. Each node is assigned a random natural frequency. The values in the horizontal axis represent the maximum possible difference between a node’s random time constant and the fixed values in S3 Table (given as a percentage of the fixed values). **a**) Number of controllable systems. **b**) Lowest energetic cost across the nodes. Given is the mean ± SD of N = 10 realizations of the time constants vectors for each of the variabilities.
(TIF)

S1 Table IDs of the ADNI subjects included in the study. (DOCX)

S2 Table Demographic characteristics of the 41 ADNI subjects included in the study. (DOCX)

S3 Table Values of the parameters used. (DOCX)

S1 Text Optimal control theory and control matrices. (DOCX)

S2 Text Network topological measures. (DOCX)

S3 Text The effect of modeling with different natural frequencies on the performance of the controllers. (DOCX)
