# Supplementary figures and images for: Design of optimal nonlinear network controllers for Alzheimer's disease
Source: PLoS Comput Biol. 2018 May 24;14(5):e1006136. doi: 10.1371/journal.pcbi.1006136 (PMC5967700; doi:10.1371/journal.pcbi.1006136)

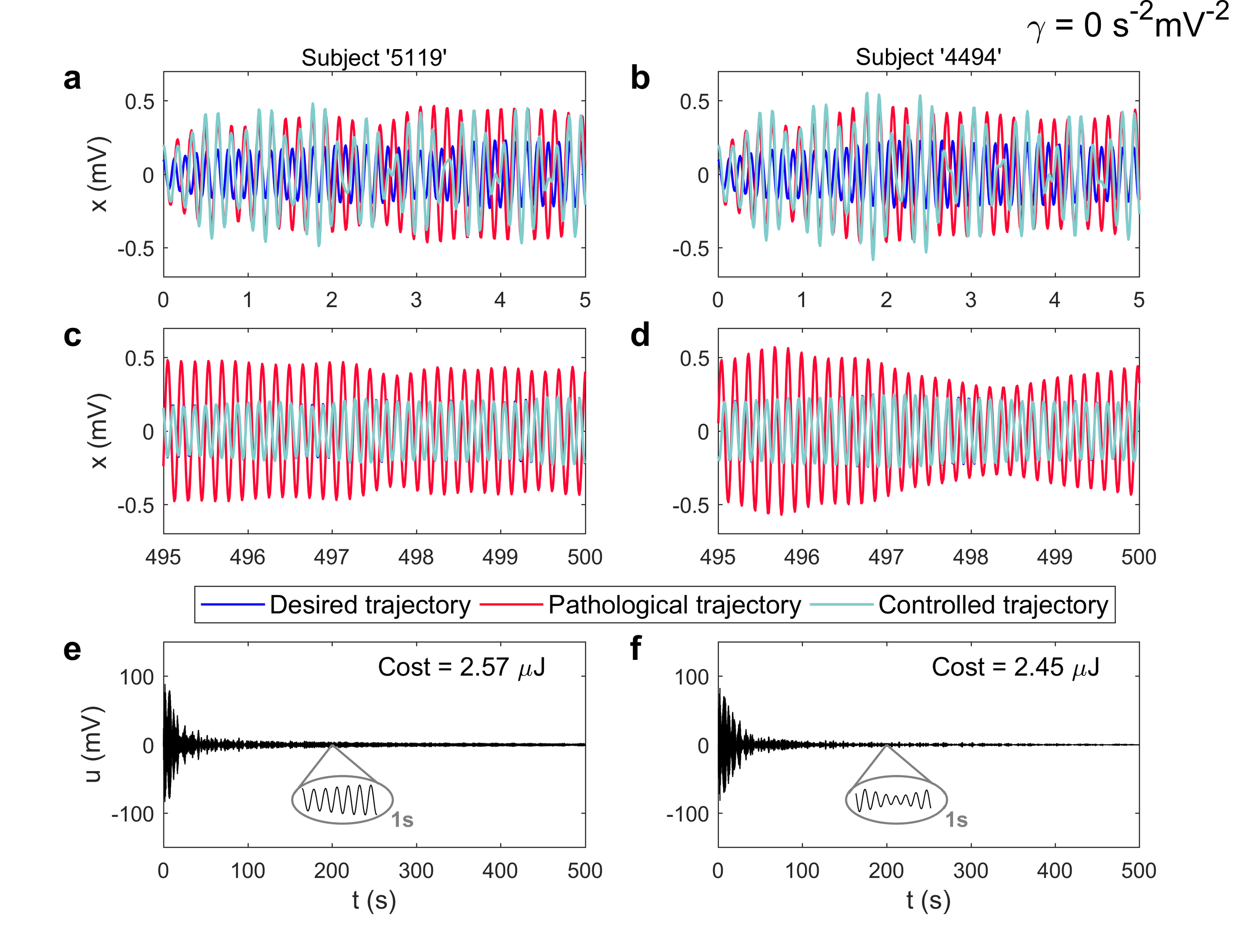

Supplement: S1 Fig — a) Start of the simulations for the ADNI subject identified as ‘5119’. The evolution of the postsynaptic potential over one region is shown only. Others behave analogously. The desired trajectory corresponds to a ‘healthy’ low-amplitude alpha-band oscillation. The model can also produce ‘pathological’ high-amplitude theta-band oscillations. A control signal feeds the left pallidum for reverting the pathological activity. c) By the end of the simulation, the controlled trajectory almost identically matches the healthy state although it was created with the ‘pathological parameters’. This is the effect of the optimal control signal, shown in (e). Panels (b,d,f) present the same analysis for the subject identified as ‘4494’. The energetic cost of the control task is inserted in (e) and (f). A one-second zoom-in window of the control signal at t = 200s is also inserted. The strength of the nonlinearity was set to γ = 0 s−2mV−2. This figure is the linear systems-equivalent to Fig 2 in the main text. (TIF) [file pcbi.1006136.s001.tif]

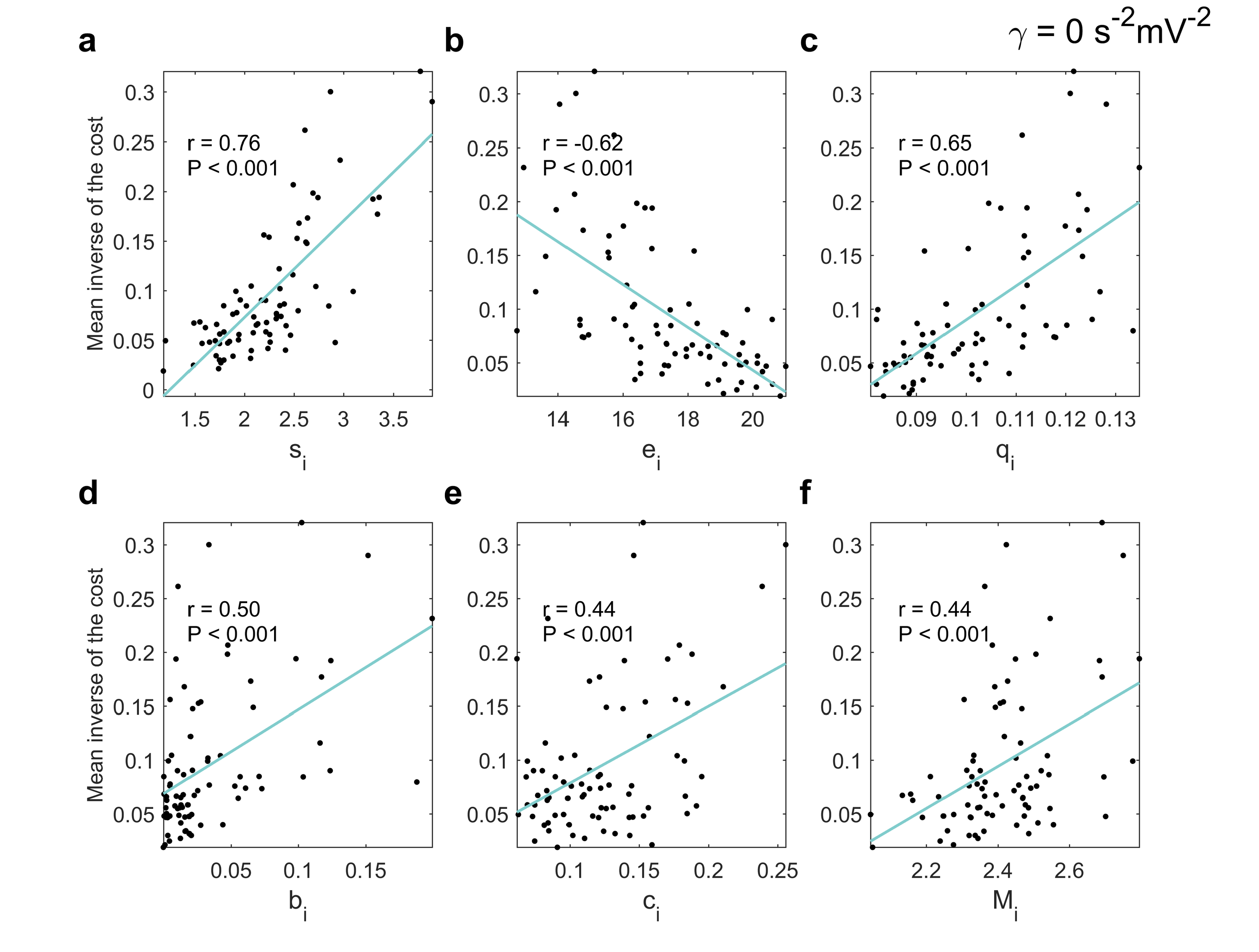

Supplement: S2 Fig — Relationship between the mean inverse of the cost across the sample and the mean node strength (a) (linear regression: F(1,76) = 105.72, P < 0.001), eccentricity (b) (linear regression: F(1,76) = 46.58, P < 0.001), closeness centrality (c) (linear regression: F(1,76) = 55.70, P < 0.001), betweenness centrality (d) (linear regression: F(1,76) = 25.20, P < 0.001), clustering coefficient (e) (linear regression: F(1,76) = 18.70, P < 0.001) and communicability (f) (linear regression: F(1,76) = 18.26, P < 0.001); N = 78 regions, in all cases. The Pearson correlation coefficients, r, are inserted. The strength of the nonlinearity was set to γ = 0 s−2mV−2. This figure is the linear systems-equivalent to Fig 5 in the main text. (TIF) [file pcbi.1006136.s002.tif]

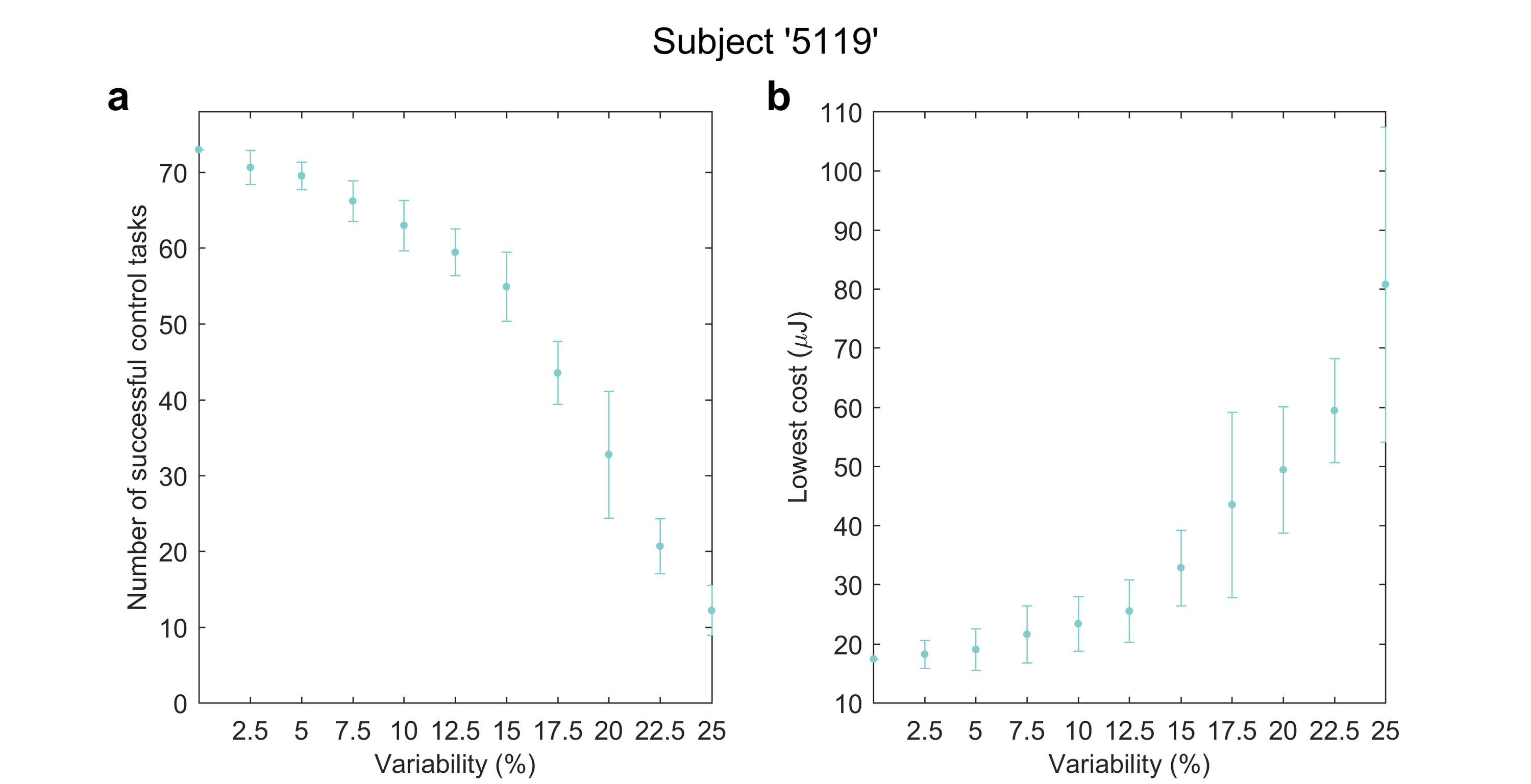

Supplement: S3 Fig — Each node is assigned a random natural frequency. The values in the horizontal axis represent the maximum possible difference between a node’s random time constant and the fixed values in S3 Table (given as a percentage of the fixed values). a) Number of controllable systems. b) Lowest energetic cost across the nodes. Given is the mean ± SD of N = 10 realizations of the time constants vectors for each of the variabilities. (TIF) [file pcbi.1006136.s003.tif]
