# Supplementary material for: Design of optimal nonlinear network controllers for Alzheimer's disease
Source: PLoS Comput Biol. 2018 May 24;14(5):e1006136. doi: 10.1371/journal.pcbi.1006136 (PMC5967700; doi:10.1371/journal.pcbi.1006136)
Supplement: S1 Table — (DOCX) [file pcbi.1006136.s004.docx]

**S1 Table**. **IDs of the ADNI subjects included in the study**

| **Subjects’ IDs** |
| --- |
| ‘4136’; ‘4152’; ‘4373’; ‘4892’; ‘4707’; ‘4910’; ‘5038’; ‘5119’; ‘4568’; ‘4911’; ‘4009’; ‘4583’; ‘4591’; ‘4887’; ‘4963’; ‘5032’; ‘5057’; ‘4718’; ‘4924’; ‘4801’; ‘4802’; ‘4938’; ‘4962’; ‘4964’; ‘4307’; ‘4959’; ‘5062’; ‘4089’; ‘4282’; ‘4737’; ‘4201’; ‘4215’; ‘4494’; ‘4686’; ‘4500’; ‘4940’; ‘4992’; ‘5028’; ‘5056’; ‘5058’; ‘5067’. |
