# Supplementary material for: Design of optimal nonlinear network controllers for Alzheimer's disease
Source: PLoS Comput Biol. 2018 May 24;14(5):e1006136. doi: 10.1371/journal.pcbi.1006136 (PMC5967700; doi:10.1371/journal.pcbi.1006136)
Supplement: S2 Table — (DOCX) [file pcbi.1006136.s005.docx]

**S2 Table**. **Demographic characteristics of the 41 ADNI subjects included in the study**

| **Characteristic** | Females | Age(years) | Education(years) | APOE e4 (1 copy) | APOE e4 (2 copies) |
| --- | --- | --- | --- | --- | --- |
| **Values**  **(41 AD-patients)** | 14(34.1) | 75.6(8.0) | 15.3(3.0) | 20(48.7) | 5(12.2) |

Data appears as mean (SD) or number of subjects (percentage)
