# Supplementary material for: Design of optimal nonlinear network controllers for Alzheimer's disease
Source: PLoS Comput Biol. 2018 May 24;14(5):e1006136. doi: 10.1371/journal.pcbi.1006136 (PMC5967700; doi:10.1371/journal.pcbi.1006136)
Supplement: S3 Table — (DOCX) [file pcbi.1006136.s006.docx]

**S3 Table**. **Values of the parameters used.**

| **Parameter** | **Description** | **Value, units** |
| --- | --- | --- |
| $\alpha_{p}$ | ‘Time constant’ for the pathological system | $1935 s^{-2}$ |
| $\alpha_{h}$ | ‘Time constant’ for the healthy system | $2852 s^{-2}$ |
| $\beta$ | Global coupling strength | $150 s^{-2}$ |
| $\gamma$ | Strength of the nonlinearity | $\left[ 0:50:300 \right] s^{-2}{mV}^{-2}$ |
| $\left( \boldsymbol{x}_{0}, \boldsymbol{y}_{0} \right)_{p}$ | Initial conditions for the pathological system | $\left[ 0.2\cdot\boldsymbol{1}_{N\times1} mV\boldsymbol{;}\boldsymbol{0}_{N\times1} s^{-1}mV \right]$ |
| $\left( \boldsymbol{x}_{0}, \boldsymbol{y}_{0} \right)_{h}$ | Initial conditions for the healthy system | $\left[ 0.1\cdot\boldsymbol{1}_{N\times1} mV\boldsymbol{;}\boldsymbol{0}_{N\times1} s^{-1}mV \right]$ |

The values for the global coupling strength, $\beta$, and the initial conditions are set to produce EEG-like activity [1,2]. In the same way, the ‘time constants’, $\alpha_{h}$ and $\alpha_{p}$, are fixed a priori across all nodes, to obtain oscillations at approximately$6.4Hz$ (pathological state) and $8.0Hz$ (healthy state) [3]. These constants are within the range corresponding to theta-alpha activity [4]. The control tasks were assessed for strengths of the nonlinearity, $\gamma$, from 0 to 300, with incremented step size of 50.

S3 Table. Supplementary references

1. Jansen BH, Rit VG. Electroencephalogram and visual evoked potential generation in a mathematical model of coupled cortical columns. Biol Cybern [Internet]. 1995 Sep;73(4):357–66. Available from: https://link.springer.com/article/10.1007/BF00199471

2. Cveticanin L. Analysis Techniques for the Various Forms of the Duffing Equation. In: The Duffing Equation: Nonlinear Oscillators and their Behaviour [Internet]. Chichester, UK: John Wiley & Sons, Ltd; 2011. p. 81–137. Available from: http://doi.wiley.com/10.1002/9780470977859.ch4

3. Bennys K, Rondouin G, Vergnes C, Touchon J. Diagnostic value of quantitative EEG in Alzheimer’s disease. Neurophysiol Clin Neurophysiol. 2001;31(3):153–60.

4. Zavaglia M, Astolfi L, Babiloni F, Ursino M. A neural mass model for the simulation of cortical activity estimated from high resolution EEG during cognitive or motor tasks. J Neurosci Methods. 2006;157(2):317–29.
