# Supplementary material for: Design of optimal nonlinear network controllers for Alzheimer's disease
Source: PLoS Comput Biol. 2018 May 24;14(5):e1006136. doi: 10.1371/journal.pcbi.1006136 (PMC5967700; doi:10.1371/journal.pcbi.1006136)
Supplement: S1 Text — (DOCX) [file pcbi.1006136.s007.docx]

**S1 Text. Optimal control theory and control matrices**

Let us assume we have a generic $n-$dimensional dynamical system as:

$\dot{\boldsymbol{e}}\left( t \right)=\boldsymbol{f}\left( \boldsymbol{e} \right)\boldsymbol{+B}u\left( t \right)\boldsymbol{;e}\left( 0 \right)\boldsymbol{=}\boldsymbol{e}_{0}$**,** (S1.1)

If the drift term, $\boldsymbol{f}$, satisfies that i) $\boldsymbol{f}\left( \boldsymbol{e} \right)\boldsymbol{\in}C^{k},k\geq1$ (being $C^{k}$ the set of functions with continuous derivatives up to the $k-$order) and ii) $\boldsymbol{f}\left( 0 \right)\boldsymbol{=}0$, it can be re-written as the product of the state vector and a matrix that depends on the state itself, $\boldsymbol{f}\left( \boldsymbol{e} \right)\boldsymbol{=A}\left( \boldsymbol{e} \right)\boldsymbol{e}$ [1].

Taking equation (1) in the main document, two systems are constructed with identical parameters except for $\alpha$ (${\alpha=\alpha}_{p}$ and ${\alpha=\alpha}_{h}$) and an external input entering the pathological system ($\alpha_{p}$) for steering it towards the healthy state ($\alpha_{h}$). Equation (S1.1) is obtained from defining $\boldsymbol{e=z}_{p}\boldsymbol{-}\boldsymbol{z}_{h}$ and using $\left( a-b \right)^{3}=a^{3}-3a^{2}b+3ab^{2}-b^{3}$ [2]. A convenient selection [1] of matrix $\boldsymbol{A}\left( \boldsymbol{e} \right)$ for this system is:

$\boldsymbol{A}\left( \boldsymbol{e} \right)\boldsymbol{=}\left[ \begin{matrix} \boldsymbol{0}_{N\times N} & \boldsymbol{I}_{N\times N} & \boldsymbol{0}_{N\times1} \\ \tilde{\boldsymbol{A}} & \boldsymbol{0}_{N\times N} & \left\{ \frac{\boldsymbol{-}\left( \alpha_{p}-\alpha_{h} \right)\boldsymbol{x}_{h}}{v} \right\} \\ \boldsymbol{0}_{1\times N} & \boldsymbol{0}_{1\times N} & \boldsymbol{-}\lambda\end{matrix} \right]$**,** (S1.2)

$\tilde{A}_{ji}\boldsymbol{=}\left\{ \begin{aligned} {-\alpha}_{p}\boldsymbol{-}\gamma\left( e_{\boldsymbol{x}i}^{2}+3e_{\boldsymbol{x}i}{x_{h}}_{i}+3{{x_{h}}_{i}}^{2} \right) i=j \\ \beta W_{ji} otherwise \end{aligned} \right.$

where the system has also been augmented with a new equation for a stable state $v\left( t \right)$ as a workaround solution to overcome the presence of state-independent terms [3]:

$\dot{v}\left( t \right)=-\lambda v\left( t \right), v\left( 0 \right)=1, \lambda=1$**,** (S1.3)

The symbol $e_{\boldsymbol{x}i}$ represents the $i-$th difference of the pathological and healthy solutions in the observable variables only, $e_{\boldsymbol{x}i}=\left( \boldsymbol{x}_{p}-\boldsymbol{x}_{h} \right)_{i}$.

Now, under these transformations, (S1.1) has a (apparent) linear form and the linear quadratic control theory can be used to obtain the optimal control signal, $u\left( t \right)$ [1,4]. In regulator problems, the system is required to maintain a steady state. A quadratic cost index is to be minimized in a time interval far bigger than the system’s time scales (infinite time):

$J=\frac{1}{2}\int_{0}^{\infty} \left[ {\boldsymbol{e}\left( t \right)}^{T}\boldsymbol{Qe}\left( t \right)\boldsymbol{+}{u\left( t \right)}^{T}\boldsymbol{R}u\left( t \right) \right]dt$**,** (S1.4)

The weight matrices $\boldsymbol{Q}$ and $\boldsymbol{R}$ are chosen based on the speed of the responses and distance from the equilibrium point –the origin– that are sought to be achieved by the controller [5]. The second term in the integral associates with the energy used by the controller [6]. Additionally, observability is guaranteed if the matrix $\boldsymbol{Q}$ is positive definite. In this work, we chose $\boldsymbol{R=}1$ and $\boldsymbol{Q}$ is a matrix of zeros except for the upper left $\frac{n}{2}\times\frac{n}{2}-$submatrix, which is the identity matrix (according to the definitions in S3 Table, the values in $\boldsymbol{R}$ and $\boldsymbol{Q}$ are expressed in $\Omega^{-1}$ and the cost, $J$, in $\mu J$). If the (augmented) system given by equations (S1.1)-(S1.3) is controllable and observable, there exists a local asymptotically stable solution to the optimal control problem [1,2]. The optimal state-feedback controller is obtained in the form:

$\boldsymbol{u}\left( t \right)\boldsymbol{=-}\boldsymbol{R}^{\boldsymbol{-}1}\boldsymbol{B}^{T}\boldsymbol{S}\left( \boldsymbol{e} \right)\boldsymbol{e}$, (S1.5)

where $\boldsymbol{S}\left( \boldsymbol{e} \right)$ is the solution to the state-dependent Riccati equation (SDRE):

$\boldsymbol{S}\left( \boldsymbol{e} \right)\boldsymbol{A}\left( \boldsymbol{e} \right)\boldsymbol{+}\boldsymbol{A}^{T}\left( \boldsymbol{e} \right)\boldsymbol{S}\left( \boldsymbol{e} \right)\boldsymbol{-S}\left( \boldsymbol{e} \right)\boldsymbol{B}\boldsymbol{R}^{\boldsymbol{-}1}\boldsymbol{B}^{T}\boldsymbol{S}\left( \boldsymbol{e} \right)\boldsymbol{+Q=0}$, (S1.6)

S1 Text. Supplementary references

1. Çimen T. State-Dependent Riccati Equation (SDRE) Control: A Survey. IFAC Proc Vol [Internet]. 2008;41(2):3761–75. Available from: https://doi.org/10.3182/20080706-5-KR-1001.00635

2. Jayaram A, Tadi M. Synchronization of chaotic systems based on SDRE method. Chaos, Solitons & Fractals [Internet]. 2006;28(3):707–15. Available from: http://linkinghub.elsevier.com/retrieve/pii/S096007790500442X

3. Cloutier JR, Stansbery DT. Nonlinear, Hybrid Bank-to-Turn/Skid-to-Turn Missile Autopilot Design. In: AlAA Guidance, Navigation, and Control Conference [Internet]. Montreal,Canada; 2001. p. 1–11. Available from: https://arc.aiaa.org/doi/abs/10.2514/6.2001-4158

4. Wernli A, Cook G. Suboptimal control for the nonlinear quadratic regulator problem. Automatica. 1975;11(1):75–84.

5. Hendricks E, Jannerup O, Sørense PH. Linear Systems Control [Internet]. Berlin, Heidelberg: Springer Berlin Heidelberg; 2008. 555 p. Available from: http://link.springer.com/10.1007/978-3-540-78486-9

6. Betzel RF, Gu S, Medaglia JD, Pasqualetti F, Bassett DS. Optimally controlling the human connectome: the role of network topology. Sci Rep [Internet]. 2016 Nov 29;6(1):30770. Available from: https://www.nature.com/articles/srep30770.pdf
