# Supplementary material for: Design of optimal nonlinear network controllers for Alzheimer's disease
Source: PLoS Comput Biol. 2018 May 24;14(5):e1006136. doi: 10.1371/journal.pcbi.1006136 (PMC5967700; doi:10.1371/journal.pcbi.1006136)
Supplement: S2 Text — (DOCX) [file pcbi.1006136.s008.docx]

**S2 Text. Network topological measures**

A network is usually represented as a graph in which the nodes correspond to the elements and the edges symbolize the existence of an interaction between elements. In this work, we deal with undirected and weighted networks –given by the anatomical connection density matrices– which consist of a set of $N$ nodes, $\Delta$, a set of edges, $\Gamma$ and a set of weights, $W$: $G=\left[ \Delta,\Gamma,W \right]$. A weight, $W_{ji}$, represents the fraction of a region’s surface involved in the axonal connection with respect to the total surface of both regions, $i$ and $j$. We assume that the physical length of an edge connecting $i$ and $j$ is inversely proportional to $W_{ji}$ (areas with high ‘connectivities’ are physically closer). Thus, the shortest weighted path length between any two nodes in the graph, $l_{ji}^{\omega}$, is their shortest weighted (geodesic) distance [1]. Several quantities characterize the connectivity profiles of the elements and the networks altogether. Below, we briefly define the measures we use in this work [2–5].

*Local measures*

Strength ($s_{i}$): the sum of the weights of the edges connected to node $i$:

$s_{i}=\sum_{j\in G} W_{ji}$, (S2.1)

Eccentricity ($e_{i}$): the maximal shortest path length between node $i$ and any other node in the graph:

$e_{i}=\max_{j\in G,j\neq i} l_{ji}^{\omega}$, (S2.2)

Closeness centrality ($q_{i}$): the average distance between node $i$ and every other node in the graph:

$q_{i}=\frac{N-1}{\sum_{j\in G,j\neq i} l_{ji}^{\omega}}$, (S2.3)

Betweenness centrality ($b_{i}$): the fraction of all shortest paths in the network that contain node $i$:

$b_{i}=\frac{1}{\left( N-1 \right)\left( N-2 \right)}\sum_{\begin{aligned} h,j\in G \\ h\neq j\neq i \end{aligned}} \frac{\sigma_{hj}\left( i \right)}{\sigma_{hj}}$, (S2.4)

$\sigma_{hj}$ is the total number of paths from $h$ to $j$ and $\sigma_{hj}\left( i \right)$ is the number of these paths passing through node $i$.

Clustering coefficient ($c_{i}$): the fraction of triangles around node $i$ :

$c_{i}=\frac{2t_{i}^{\omega}}{k_{i}\left( k_{i}-1 \right)}$, (S2.5)

$k_{i}$ is the degree of node $i$ (total number of edges connected to it) and $t_{i}^{\omega}$ is the weighted geometric mean of triangles around $i$, $t_{i}^{\omega}=\frac{1}{2}\sum_{h,j\in G} \left( W_{ji}W_{ih}W_{hj} \right)^{\frac{1}{3}}$.

Node communicability ($M_{i}$): the communicability counts (direct and indirect) paths of all lengths between two nodes and is defined by the operation: $M_{ji}={\sum_{k=0}^{\infty} \left( \frac{\boldsymbol{H}^{k}}{k!} \right)}_{ji}$, where $\boldsymbol{H=}\mathbf{D}^{-\frac{1}{2}}\mathbf{W}\mathbf{D}^{\frac{1}{2}}$ and $\mathbf{D}\in\mathbb{R}^{N\times N}$ is the matrix with diagonal elements $D_{ii}=\sum_{j\in G} W_{ji}$. We use a node communicabilit*y* instead, which is obtained from adding the communicabilities between node $i$ and every other node in the graph. Thus:

$M_{i}=\sum_{j\in G} M_{ji}$, (S2.6)

*Global measures*

Characteristic path length ($l$): the average shortest path length in the network:

$l=\frac{1}{N}\sum_{i\in G} \frac{\sum_{j\in G,j\neq i} l_{ji}^{\omega}}{N-1}$, (S2.7)

Radius ($r$): the minimum eccentricity:

$r=\min_{i\in G} e_{i}$, (S2.8)

Diameter ($d$): the maximum eccentricity:

$d=\max_{i\in G} e_{i}$, (S2.9)

Average clustering coefficient ($C$):

$C=\frac{1}{N}\sum_{i\in G} c_{i}$, (S2.10)

Global efficiency ($E_{g}$): the average inverse shortest path length in the network:

$E_{g}=\frac{1}{N}\sum_{i\in G} \frac{\sum_{j\in G,j\neq i} \left( l_{ji}^{\omega} \right)^{-1}}{N-1}$, (S2.11)

S2 Text. Supplementary references

1. Iturria-Medina Y, Sotero RC, Canales-Rodríguez EJ, Alemán-Gómez Y, Melie-García L. Studying the human brain anatomical network via diffusion-weighted MRI and Graph Theory. Neuroimage [Internet]. 2008 Apr;40(3):1064–76. Available from: http://linkinghub.elsevier.com/retrieve/pii/S1053811907010014

2. Barrat A, Barthelemy M, Pastor-Satorras R, Vespignani A. The architecture of complex weighted networks. Proc Natl Acad Sci [Internet]. 2004 Mar 16;101(11):3747–52. Available from: http://arxiv.org/abs/cond-mat/0311416

3. Estrada E, Hatano N. Communicability in complex networks. Phys Rev E - Stat Nonlinear, Soft Matter Phys. 2008;77(3):1–12.

4. Rubinov M, Sporns O. Complex network measures of brain connectivity: Uses and interpretations. Neuroimage [Internet]. 2010;52(3):1059–69. Available from: http://dx.doi.org/10.1016/j.neuroimage.2009.10.003

5. Betzel RF, Gu S, Medaglia JD, Pasqualetti F, Bassett DS. Optimally controlling the human connectome: the role of network topology. Sci Rep [Internet]. 2016 Nov 29;6(1):30770. Available from: https://www.nature.com/articles/srep30770.pdf
