# Supplementary material for: Design of optimal nonlinear network controllers for Alzheimer's disease
Source: PLoS Comput Biol. 2018 May 24;14(5):e1006136. doi: 10.1371/journal.pcbi.1006136 (PMC5967700; doi:10.1371/journal.pcbi.1006136)
Supplement: S3 Text — (DOCX) [file pcbi.1006136.s009.docx]

**S3 Text. The effect of modeling with different natural frequencies on the performance of the controllers**

The objective of the herein described simulations was to compare the results of the controllers with different variabilities within the set of oscillators’ inner frequencies. Distinct theta and alpha time constants were obtained by adding the original unique $\alpha_{h}$ and $\alpha_{p}$ (S3 Table) to a $N\times1$- vector generated from a continuous uniform distribution with endpoint fixed to a percentage of those original values. The maximum variability in the experiment was set to force the oscillators to remain in the designated frequency band, both a priori and after their dynamical interaction. The strength of the nonlinearity was $\gamma=200 s^{-2}{mV}^{-2}$. The rest of the parameters were set to the values specified in S3 Table and S1 Text, except for $\left( \boldsymbol{x}_{0}, \boldsymbol{y}_{0} \right)_{p}= \left[ 0.15\cdot\boldsymbol{1}_{N\times1} mV\boldsymbol{;}\boldsymbol{0}_{N\times1} s^{-1}mV \right]$ and $\boldsymbol{Q}=10\cdot\left[ \begin{matrix} \boldsymbol{I}_{N\times N} & \boldsymbol{0}_{N\times\left( N+1 \right)} \\ \boldsymbol{0}_{\left( N+1 \right)\times N} & \boldsymbol{0}_{N\times\left( N+1 \right)} \end{matrix} \right]\Omega^{-1}$. The reason why these parameters were changed from their original values was to achieve comparable results over all the simulations regardless of the variability. Such design modifications, like changing the weight function $\boldsymbol{Q}$**,** are easy to implement in the scope of SDRE [1] and totally valid given that they are reflected in the obtained control signals.

S3 Fig shows the behavior of the controllers with the variability in the inner frequency of the network nodes, in terms of the number of successful control tasks and the lowest energetic cost of all stimuli applied to subject ‘5119’ (mean **±** standard deviation). The predefined control objective of steering the system towards the healthy state is fulfilled for a lower number of stimuli as the variability increases (panel a). Additionally, the lowest possible energetic cost is also affected, experiencing an increase as the oscillators’ natural frequencies are more spread (panel b). In effect, the higher the variability is, the more difficult is to optimally control the diseased system. However, a single successful stimulus –obtained through SDRE– would be sufficient for *in vivo* applications of our computational predictions. In those cases, the analysis herein shown should be replaced by a more realistic approach in which the actual oscillation frequencies are estimated from the subject’s EEG activity [2,3].

S3 Text. Supplementary references

1. Çimen T. State-Dependent Riccati Equation (SDRE) Control: A Survey. IFAC Proc Vol [Internet]. 2008;41(2):3761–75. Available from: https://doi.org/10.3182/20080706-5-KR-1001.00635

2. Jimenez JC, Ozaki T. An approximate innovation method for the estimation of diffusion processes from discrete data. J Time Ser Anal. 2005;27(1):77–97.

3. Sotero RC, Trujillo-Barreto NJ, Jiménez JC, Carbonell F, Rodríguez-Rojas R. Identification and comparison of stochastic metabolic/hemodynamic models (sMHM) for the generation of the BOLD signal. J Comput Neurosci [Internet]. 2009;26(2):251–69. Available from: http://www.ncbi.nlm.nih.gov/pubmed/18836824
